# EpICC: A Bayesian neural network model with uncertainty correction for a more accurate classification of cancer

**DOI:** 10.1038/s41598-022-18874-6

**Published:** 2022-08-26

**Authors:** Prasoon Joshi, Riddhiman Dhar

**Affiliations:** Department of Biotechnology, IIT Kharagpur, Kharagpur, West Bengal 721302 India

**Keywords:** Cancer, Computational biology and bioinformatics, Machine learning

## Abstract

Accurate classification of cancers into their types and subtypes holds the key for choosing the right treatment strategy and can greatly impact patient well-being. However, existence of large-scale variations in the molecular processes driving even a single type of cancer can make accurate classification a challenging problem. Therefore, improved and robust methods for classification are absolutely critical. Although deep learning-based methods for cancer classification have been proposed earlier, they all provide point estimates for predictions without any measure of confidence and thus, can fall short in real-world applications where key decisions are to be made based on the predictions of the classifier. Here we report a Bayesian neural network-based model for classification of cancer types as well as sub-types from transcriptomic data. This model reported a measure of confidence with each prediction through analysis of epistemic uncertainty. We incorporated an uncertainty correction step with the Bayesian network-based model to greatly enhance prediction accuracy of cancer types (> 97% accuracy) and sub-types (> 80%). Our work suggests that reporting uncertainty measure with each classification can enable more accurate and informed decision-making that can be highly valuable in clinical settings.

## Introduction

Recent explosion in genomic, epigenomic and transcriptomic data has provided us a glimpse of the extent of molecular heterogeneity in cancer and has led to classification of cancer into different types and sub-types based on their molecular signatures^1–7^. Existence of heterogeneity in cancer assumes significant importance for the therapeutic interventions. Different types and sub-types of cancer are driven by distinct molecular factors and often require specific anti-cancer treatment^8^. Therefore, an accurate classification method can greatly aid in choosing appropriate treatment strategies specifically targeted towards these types and sub-types^9–15^.

Among all the omics datasets, the transcriptomic data holds a lot of promise for classification of cancer types. This is because of the fact that diverse genomic and epigenomic changes often eventually impact the same cellular processes and this is reflected in the gene expression program of the cell^16,17^. This, in turn, can greatly enable accurate prediction of disease type and progression^18,19^. There are, however, several challenges associated with the use of transcriptomic data for classification of cancer types and sub-types. These datasets are high-dimensional and the expression values of many genes are intertwined in a highly complex manner^20^. In addition, the measurements from two different samples are rarely obtained under the same conditions, thus adding noise to the data.

In this scenario, machine learning and deep learning techniques can greatly aid in accurate classification of cancer type and sub-types as these techniques can capture the complex and non-linear relationships within the data^21^. Machine learning technique has been applied to predict inactivation of a tumor suppressor gene in glioblastoma and to predict patient response to chemotherapeutic drugs with good accuracy^22,23^. Artificial Neural Networks (ANN) or Deep Neural Networks (DNN), consisting of complex network of simple information propagating units (neurons), can learn the patterns ingrained in complex datasets and thus, are increasingly being applied for modelling complex and high dimensional biological datasets^24–27^.

Several methods based on artificial neural networks and deep learning have already been developed for classification of cancer types and they show good accuracy^24,25,28–31^. One of the first studies applied ANNs for prediction of small, round blue-cell tumors (SRBCTs) and could achieve high accuracy of prediction^28^. Further, Lyu and Haque^29^ utilized a convolutional neural network that could predict 33 different cancer types from transcriptome data with an accuracy of ~ 95%. Kim et al*.*^31^ used a neural network-based method to classify 21 different types of cancers using bulk as well as single cell RNA-Seq data and could achieve accuracy of ~ 90%. Xiao et al.^25^ developed a semi-supervised deep learning method that could predict three cancer types with accuracy varying between 96%-99%. Gao et al.^32^ devised a deep learning-based cancer classification method that could be applied to single samples with ~ 90% accuracy. However, the accuracy declined with a reduction in the number of feature genes.

However, all these methods used point estimates for model parameters. This has several drawbacks. First, this can result in overconfident decisions in case of limited data and when there is an imbalance in the number of samples of different cancer types^33^. Second, one does not have any measure of confidence in the prediction values which could be especially problematic for test data falling outside the distribution of the training dataset^34^. In addition, the eventual goal of all such cancer classification techniques is to devise a classifier that can classify individual patient samples into cancer types and sub-types. Here a measure of confidence or uncertainty with each prediction is important to ascertain the reliability of class predictions and can greatly benefit clinical decision-making^35^.

The performance of a deep learning model is heavily dependent on the quality of the dataset on which the model is trained on. Thus, uncertainty in predictions by a deep learning model can be of two different types. The first type of uncertainty is aleatoric uncertainty which arises due to quality of data where datapoints with imprecise measurements or labels are included^36,37^. The second form of uncertainty arises from choice of the type of model, and the selection of model parameters and is referred to as epistemic uncertainty^36,37^. Thus, running the same model multiple times on the same dataset can lead to different predictions. Uncertainty can be estimated through various methods. Bayesian framework is an ideal way to measure epistemic uncertainty as distributions of values for the model parameters are obtained. However, Bayesian neural networks are often computationally intractable to train and thus require approximations such as variational inference, Markov Chain Monte Carlo (MCMC) method and Laplace transformation^38–41^. In addition, Gal and Ghahramani^34^ showed that dropouts in non-Bayesian neural networks can estimate uncertainty values in predictions and these are equivalent to Bayesian inference. Further, Lakshminarayanan et al.^42^ showed that use of ensembles of neural networks can also enable estimation of predictive uncertainty.

In this work, we developed a deep learning-based cancer classification method called Epistemic Invariance in Cancer Classification (EpICC). This method utilized a Bayesian neural network (BNN) and analysed the uncertainty in the classification of cancer types and subtypes. Addressing the issue of aleatoric uncertainty requires acquiring new better-quality datasets which is beyond the scope of model fitting. Epistemic uncertainty, however, can be accounted for through model fitting. Thus, with EpICC, we incorporated a model-based correction of uncertainty at the output of the BNN that greatly enhanced classification accuracy. We applied this method to classification of 31 cancer types from their transcriptomic profiles. The BNN alone could achieve an overall accuracy of 93.7%. With the incorporation of Model-based uncertainty correction, EpICC could classify cancer types with an accuracy of 97.83%. In addition, EpICC could classify sub-types of four cancer types with an accuracy of > 80%. Thus, we believe that uncertainty correction can greatly aid in making more informed, accurate and reliable decisions. This is critical in clinical prediction tasks where an accurate prediction can significantly improve patients’ well-being.

## Results

### EpICC combines a Bayesian neural network (BNN) with uncertainty correction for cancer classification

**Figure 1:**
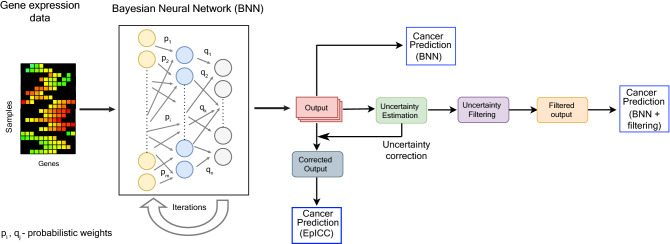
EpICC combines Bayesian Neural Network (BNN) with uncertainty correction. BNN utilizes the gene expression data of feature genes for cancer classification. The BNN consists of 3 layers with the first layer consisting 250 neurons, the second layer containing 95 neurons and the final layer consists of output neurons. The number of output neurons is dependent on the number of classes to be predicted. The weights of the connections were initialized from prior probability distributions. We refined the weights over multiple iterations through the BNN. The output was used for uncertainty estimation. After estimating the uncertainty, we tested two different approaches for incorporating uncertainty to improve classification accuracy—uncertainty filtering and uncertainty correction. We thus obtained the filtered and the corrected outputs respectively which we used for cancer type and subtype prediction.

To build a classifier that reports confidence measures associated with each prediction, we utilized a Bayesian Neural Network (BNN) for classification. The weights of the connections were determined from prior probability distributions and their values were gradually improved from learning on new data to generate posterior distributions (Fig. 1). In contrast to a typical deep neural network (DNN), BNN estimated weights in the form of probabilistic distributions and thereby could account for uncertainty in the predictions. In the method described here, we used a three-layered BNN, having 250 units in the first layer, 95 units in the hidden layer and an output layer. We chose these hyperparameters using five-fold cross-validation for best model performance. We used Bayes by Backprop algorithm^41^ for optimization of the model parameters.

BNN, in addition to providing regularization, paved the way for the analysis of uncertainty of the predictions. We added a level of confidence with each prediction through modelling Epistemic uncertainty^43^ that arose due to variations in the model structure and parameters. Since the Bayesian approach in neural networks was not computationally tractable^44^, we utilized variational distribution or variational posterior that was assumed to approximate the true posterior. This was done by minimizing the Kullback–Leibler (KL) divergence^45^ between the variational posterior and the true posterior.

We estimated epistemic uncertainty by performing multiple iterations of testing and quantifying the variation in the predictions. To do so, during inference, we performed Monte Carlo sampling of weights from the approximate variational distribution and obtained the prediction class. We repeated this over 500 iterations and calculated the variation in output by estimating the average of the difference between the actual softmax probability in individual iterations and the mean softmax probability over all iterations (see Methods). This gave us the uncertainty values for predictions of all individual classes (Fig. 1).

Uncertainty enabled us to reduce incorrect predictions in cancer classification. We tested two approaches incorporating uncertainty for improving cancer classification. First, in the filtering approach, we chose a threshold in the uncertainty value obtained as above for distinguishing between correct and incorrect predictions and discarded all predictions showing higher uncertainty than the threshold as wrong predictions (Fig. 1). However, this resulted in a decrease in the number of samples on which predictions were made as several samples were discarded. To address this drawback, we introduced a second uncertainty correction approach where we performed a model-based uncertainty correction at the output of the Bayesian Neural Network. To do so, for each cancer type, we fitted a linear model between the log odds ratio of the expected value of the predicted output from Monte Carlo iterations and the square root of epistemic uncertainty. We then used Ordinary Least Squares (OLS) to calculate the coefficients of the linear model. This enabled us to calculate the corrected prediction probabilities for each cancer class (Fig. 1).

### EpICC classifies 31 different cancer types from gene expression profile with high accuracy

We applied EpICC for classification of 31 different cancer types from > 10,000 cancer samples for which we obtained the expression profiles from The Cancer Genome Atlas (TCGA) data portal. We first applied a two-step principal component analysis (PCA) and logistic regression to select the genes (or features) that had the highest power to distinguish different cancer types from the transcriptomic data (Supplementary fig. S1). We identified 103 marker genes in this process. Selecting genes in two steps enabled us to identify genes that could explain a greater percentage of variance in the data than those identified in only one step (Supplementary fig. S1D). These 103 marker genes were able to distinguish between normal tissue and cancer tissue from their expression profiles with 100% accuracy, suggesting that these genes are likely to be important markers for identification of cancer state.

**Figure 2:**
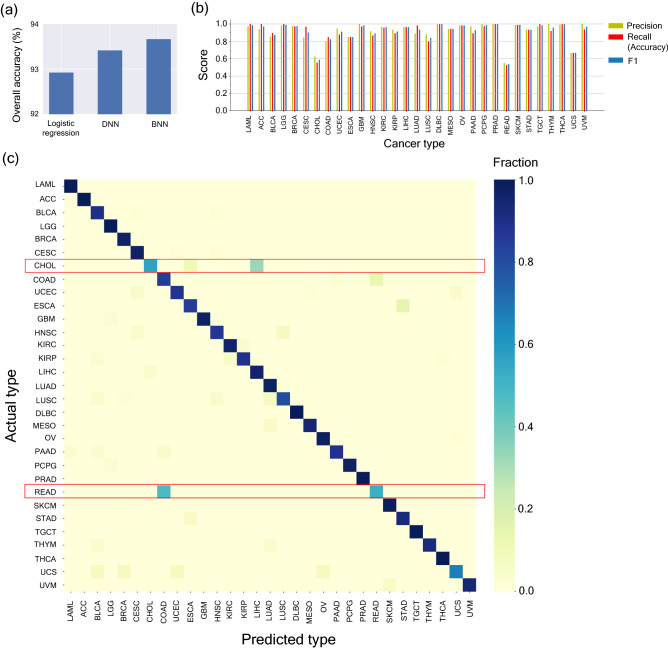
Classification of 31 different cancer types by a Bayesian neural network (BNN) (**a**) Overall Accuracy comparison of L2-regularized logistic regression, L2-regularized Deep Neural Network based classification method (DNN) and Bayesian Neural Network based classification method (BNN) (**b**) Precision, recall and F1 Scores of the cancer types for prediction of individual cancer types by the BNN. For classification of individual cancer types, recall represents accuracy of classification. (**c**) Confusion Matrix for the predictions of individual cancer types by BNN. The rows denote the actual cancer types and the columns denote the predicted cancer types.

We next investigated whether these 103 genes (features) (Supplementary table S1) could accurately classify 31 different cancer types (Supplementary table S2) from their transcriptomic profiles. In addition to applying the BNN method, we also classified the data using an L2 regularized DNN model and an L2-regularized logistic regression model for performance comparison. BNN gave an overall accuracy of 93.66%, which was similar to the overall accuracy of 93.41% for DNN and was marginally higher than the overall accuracy of 92.82% for logistic regression (Fig. 2a).

In addition to the overall accuracy, we also quantified the true positive, false positive, true negative and false negative rates using precision, recall and F1 score for different cancer types (Fig. 2b,c). For classification of individual cancer types, recall value represents accuracy and the F1 score (harmonic mean of precision and recall) provides a combined picture of the overall specificity and accuracy of the classification method. Individual F1 Scores for all cancer types were greater than 0.80 except for Cholangiocarcinoma, CHOL (F1 Score = 0.59), Rectum Adenocarcinoma, READ, (F1 Score = 0.54) and Uterine Carcinosarcoma, UCS, (F1 Score = 0.67) (Fig. 2c). Analysis of confusion matrix revealed that READ was falsely classified as COAD almost 48% of the times and CHOL was falsely classified as LIHC almost 33% of the times (Fig. 2c). The most likely reason for such mis-classifications could be the close proximity of these organs which could lead to sample contamination^46^ as well as low number of available samples for these cancer types (Supplementary table S3).

Almost 85% of the 103 feature genes in our analysis were associated with either oncogenic function or tumor suppressor function in different cancer types or were reported biomarkers across at least one cancer type (Supplementary table S1). Approximately ~ 39% of the genes were already reported to have oncogenic activity across different cancer types and ~ 61% of genes reported were earlier associated with at least one type of cancer or were used as a biomarker. We further tested whether expression pattern of a small subset of these 103 genes (Supplementary fig. S2) could predict cancer types through evaluating performance of each individual gene in cancer type classification (Supplementary fig. S3). Interestingly, we observed that single gene expression profile could correctly classify two cancer types (Supplementary fig. S4a, S4b). In addition, we could classify 10 cancer types with expression profiles of just 12 genes with Precision and Recall values greater than 0.75 (Supplementary fig. S4c).

**Figure 3:**
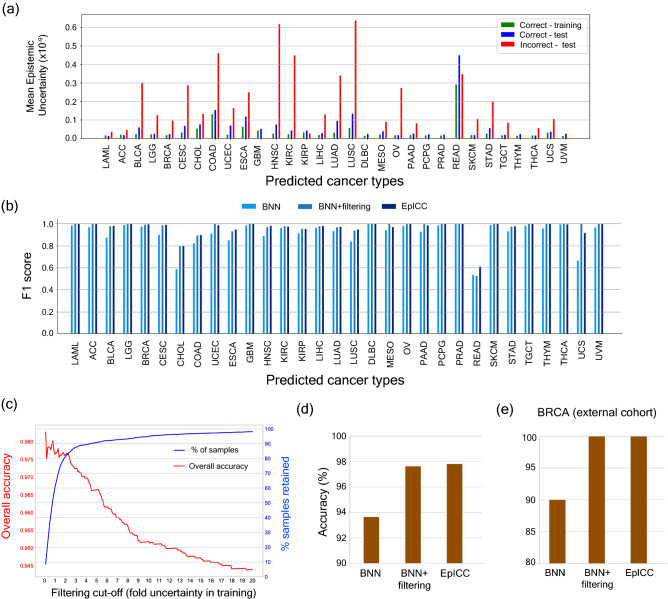
Cancer classification accuracy improves with uncertainty correction. (**a**) Plots showing the comparison of uncertainty on the correct and incorrect predictions. The green-coloured bars represent the mean uncertainty of correct predictions on training data, the blue bars show the mean uncertainty of correct predictions on test data and the red bars show the mean uncertainty of incorrect predictions on the test data. (**b**) Comparison of F1 scores of the prediction made by BNN, after filtering the predictions based on mean training uncertainties of correct predictions and after applying uncertainty correction (EpICC). (**c**) Variation in overall accuracy and the percentage of samples retained for prediction using different values of filtering cut-off. (**d**) Comparison of overall classification accuracy of BNN, BNN with uncertainty filtering and EpICC for classification of cancer types. (**e**) Percentage accuracy of classification of BRCA from external cohort (ICGC) with BNN, BNN with uncertainty filtering by mean training correct predictions, and by EpICC.

In the next step, we focused on associating confidence measures with all class predictions. To do so, we calculated the epistemic uncertainties in predictions on training and test data sets. This generated the overall uncertainty (Supplementary fig S5) across all cancer types as well as the uncertainty values for each cancer type (Fig. 3a). Incorrect classifications generally had higher uncertainty values associated with them as compared to correct classifications (Fig. 3a). Anomalies were observed in case of READ and KIRP, where the uncertainty of correct predictions were higher than that of incorrect predictions. KIRP has a high precision value (> 0.95) which means that almost all the samples classified as KIRP were correct. However, this was not the case for READ and we observed a comparatively higher percentage of false positives.

We tested two approaches utilizing uncertainty values to reduce errors—uncertainty filtering and uncertainty correction. For uncertainty filtering approach, we chose the mean epistemic uncertainty for correct classifications in the training data as the threshold for distinguishing correct and incorrect classifications in the test dataset. We considered only those classifications that had uncertainty lower than the uncertainty obtained for training data and thereby, discarded the classifications with high uncertainty. Filtering improved the overall accuracy of classification from 93.67% to 97.68%. In addition, the F1 score of classification of each cancer type improved as well (Fig. 3b). However, this resulted in a significant decline in the number of samples that were included in classification (Fig. 3c; Supplementary fig. S5). To achieve 97.65% accuracy in classification, filtering dropped ~ 40% of samples from the whole data.

This is where the second approach involving uncertainty correction proved valuable as it improved accuracy of classifications without dropping any sample from the analysis. For classification of cancer types, uncertainty correction resulted in overall accuracy of 97.83**%** without dropping any sample and also improved the F1 scores for classification of all cancer types (Fig. 3b,d).

For an independent validation of our classification method, we tested our model on the Breast Cancer Data from South Korean cohort available from ICGC^47^. At the time of this study, only this data had the same normalization as the TCGA data on which the model was trained. The number of samples were 50. We also compared the performances of BNN with Logistic Regression and a typical DNN. Bayesian Neural Network and DNN were able to classify 90% of the samples correctly, followed by Logistic Regression which was able to classify 76% of the samples correctly. Following uncertainty filtering the accuracy increased to 100% (Fig. 3e), however only 42% of the samples were retained for prediction. In contrast, uncertainty correction improved classification accuracy to 100% without dropping any samples (Fig. 3e).

### EpICC classifies cancer subtypes with high accuracy

**Figure 4:**
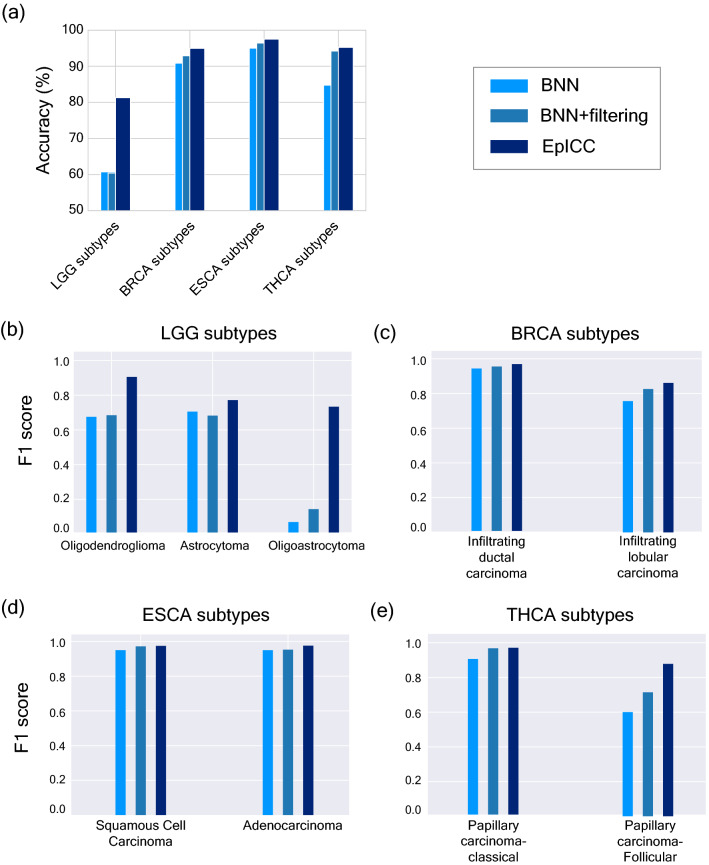
EpICC accurately predicts cancer subtypes. (**a**) Comparison of classification accuracy of BNN alone, BNN with uncertainty filtering and EpICC for cancer subtype classification. Comparison of F1 scores of the prediction made by BNN, after filtering based on mean training uncertainties of correct predictions and after applying uncertainty correction (EpICC) for (**b**) LGG subtypes (**c**) BRCA subtypes (**d**) ESCA subtypes (**e**) THCA subtypes.

With EpICC displaying high accuracy in classification of cancer types, we were also interested in testing whether EpICC could accurately classify cancer subtypes within a cancer type. Accurate classification of subtypes is often extremely crucial for deciding the precise treatment strategy. To test the predictive ability of EpICC for cancer subtypes, we collected the gene expression values of histological subtypes of LGG, BRCA, ESCA, and THCA. Only for these four cancer types, data from > 50 samples were available for each of the histological subtypes within each cancer type. We predicted the subtypes first using a simple BNN, then applied filtering and uncertainty correction method. BNN alone could achieve a test accuracy of 60% for classification of LGG subtypes, 90% for classification of BRCA subtypes, 95% for ESCA subtypes and 84.37% for THCA subtypes (Fig. 4a).

We also estimated uncertainty associated with each subtype prediction for training as well as test datasets (Supplementary fig. S6). Again, uncertainty filtering substantially improved classification accuracy and F1 score for all subtypes (Fig. 4) but led to dropping of substantial number of samples (Supplementary fig. S7). For example, in the classification of LGG subtypes, only ~ 45% of the samples were included in analysis and the rest were discarded. For classification of subtypes of the other cancer types, less than 70% of the samples were included. On the other hand, EpICC with uncertainty correction led to substantial improvement in classification accuracy (> 80%) for subtype classification across all cancer types without discarding any sample. Similarly, the F1 score for subtype classifications improved across all cancer types (Fig. 4b–e). Biggest improvement was seen in classification of LGG subtype Oligodendroglioma where the F1 score improved to 0.73 using EpICC from F1 scores of 0.07 by applying only BNN and 0.14 by applying BNN along with uncertainty filtering (Fig. 4b).

### Performance comparison of EpICC with published methods

We also benchmarked the performance of EpICC against eight published methods as shown in Table 1. The accuracy obtained using EpICC for classification of cancer types was highest among all methods that classified a substantial number of cancer types^29,31,48,49^. Only the method reported^25^ had higher accuracy than EpICC, however, it was obtained for classification of only three cancer types. Further, we also compared the accuracy of EpICC in classification of cancer subtypes within four cancer types. For the classification of LGG subtypes, EpICC was substantially better with an accuracy of ~ 81% compared to ~ 60% obtained by Pei et al.^50^. For classification of BRCA subtypes, EpICC had comparable accuracy to the method described by Couture et al.^51^. In addition, EpICC also performed very well in classification of subtypes within ESCA and THCA cancer types with accuracies over 95%.

**Table 1:** Accuracy of EpICC for classification of cancer types and sub-types in comparison to published methods.

Study	Classification accuracy (%)				
	Cancer types	LGG subtypes	BRCA subtypes	ESCA subtypes	THCA subtypes
Lyu and Haque^29^	95.59% (33)	NA	NA	NA	NA
Kim et al.^31^	91.74% (21)	NA	NA	NA	NA
Xiao et al.^25^	96%-99% (3)	NA	NA	NA	NA
Ramirez et al.^49^	94.70% (33)	NA	NA	NA	NA
Sun et al.^48^	97.47% (12)	NA	NA	NA	NA
Pei et al.^50^	NA	63.90 (3)	NA	NA	NA
Couture et al.^51^	NA	NA	94 (2)	NA	NA
EpICC	97.83% (31)	81.31 (3)	94.98 (2)	97.5 (3)	95.24 (2)

The number within the brackets show the number of cancer types or subtypes for which classifications were done.

## Discussion

In the current work, we developed a Bayesian neural network-based classifier called EpICC that was able to classify cancer types and subtypes with high accuracy. We were able to estimate epistemic uncertainty associated with each classification. Epistemic uncertainty is the variations in the classification that arises out of variations in model fitting and is the major source of uncertainty in classification tasks. Filtering our predictions by removing predictions with high uncertainty could improve our overall prediction performance. However, this also resulted in discarding of a large number of samples for which uncertainty values were higher than the cut-off values.

Therefore, we devised an uncertainty correction method that reported a corrected probability value for each classification after accounting for uncertainty. This greatly improved accuracy and did not discard any sample. In addition, we also evaluated the performance of EpICC in classification of cancer sub-types across four different cancer types. Indeed, EpICC could also classify cancer subtypes with high accuracy. We also benchmarked the performance of EpICC against already published classification methods. EpICC showed good versatility for classification of cancer types and subtypes as compared to the other methods which were applied only to cancer type classification or were limited to classification of subtypes within just one cancer type.

We could classify subtypes for only four types of cancers due to lack of enough available data for sub-types. In addition, we could apply subtype classification for prediction of histological subtypes and could not apply our method to molecular subtype classification, as not enough data was available. Further, the accuracy of subtype classification was slightly lower compared to cancer type classification. This could be due to higher overall expression similarity between sub-types of a cancer compared to similarity in expression pattern among different cancer types. This could make the classification process even more challenging. Therefore, one possibility to increase subtype classification accuracy would be to combine transcriptome data with epigenetic modification patterns in cancers. In addition, it remains to be seen whether using multi-omics datasets could enable better classification of cancer sub-types.

Taken together, the present work demonstrates the value of modelling uncertainty in cancer classification. Accounting for uncertainty not only increases accuracy of predictions but also enables us to make more informed predictions that can be tuned based on the specific requirements of different application scenarios. This can help devise a two-stage classification process where predictions characterised with high uncertainties can be further tested by additional techniques. This framework can be further expanded to classification of other cancer sub-types when more data become available. In addition, this framework holds great promise for detection of cancer types and sub-types from transcriptome data obtained from blood samples^52,53^ and can enable accurate classification of cancer from liquid biopsies as more data become available. Finally, the framework developed here can be adapted to other classification tasks where a measure of confidence can improve decision-making ability.

## Methods

### Data

The cancer data comprised of transcriptome data from 31 different cancer types and subtypes of four different cancer types measured using Illumina HiSeq 2000 RNA Sequencing platform downloaded from UCSC XENA^54^ repository. The data is characterised as level 3 data from TCGA consortium^55^ and consists of $\log_{2} \left( {x + 1} \right)$ transformed RSEM normalized counts^56^. The cancer types and their corresponding abbreviations, and the subtypes are shown in Table S1. In total, there were 10,013 cancer samples. 80% of the data was kept for training and feature selection and the remaining 20% data was reserved for testing. Using the training data, five-fold cross validation was used to tune the model hyperparameters. The training and testing data contained identical distributions of the cancer types.

To perform cancer vs non-cancer classification, the gene expression values of normal samples from the GTEX^57^ consortium in the UCSC XENA repository was downloaded. This data, too consisted of $\log_{2} \left( {x + 1} \right)$ transformed RSEM normalized counts. In total, there were 7851 normal samples. Similar 80:20 splitting of data was performed and the split data was combined with the respective TCGA data for classification. We dropped the expression values of the genes *C19orf46*, *MOSC2, LASS3, TARP, GOLGA2B, EFCAB4A* and *RTDR1* from cancer samples as the normal data did not contain expression values for these genes. L2-regularized Logistic Regression was applied for classification and 100% accuracy was obtained for classification of normal and cancer samples.

For cancer subtype analysis, the gene expression data of each cancer type used in this study was characterized into their respective subtypes, with the help of phenotypic information available in UCSC Xena repository. For every cancer type, only the subtypes that had overall 50 samples at the time of analysis were chosen so that the training and the test samples were well represented. Using this strategy, three subtypes of LGG (Oligodendroglioma. Oligoastrocytoma and the Oligoastrocytoma), two subtypes of BRCA (Invasive Ductal Carcinoma and Invasive Lobular Carcinoma), two subtypes of ESCA (Adenocarcinoma and Squamous Cell Carcinoma), and two subtypes of THCA (Papillary Cell Carcinoma and Follicular Cell Carcinoma) were selected. For each cancer type chosen for subtype classification, separate feature genes were identified by performing PCA analysis. In this case too, 80% of the data was used for feature selection, hyperparameter tuning, and training while 20% data was used for testing.

### Feature selection

To reduce the risk of overfitting and to get rid of redundant genes, Principal Components Analysis (PCA) was used on the training split of the TCGA data. This was done in two steps: (i) Selecting a set of genes from the original high dimensional RNA-seq data (ii) Selecting an even smaller set of genes from the gene set selected in the first step. To determine the number of optimal genes to be selected in steps (i) and (ii), a supervised feature selection technique was used using a combination of Principal Components Analysis (PCA) and Logistic Regression. For each component and up to 10 components, a certain number of genes having the highest absolute value of factor loadings were selected. The optimal number of genes selected in the first and the second steps were determined by Logistic Regression, which was used as a classifier to identify the minimum number of genes required to achieve a high accuracy, beyond which the accuracy did not increase much with further addition of the number of genes.

### Bayesian neural network

A typical Deep Neural Network (DNN), if viewed probabilistically, can be considered as a maximum likelihood estimate of the model parameters $w,$ where the objective is to learn the parameters such that the probability of occurrence of data given the model parameters is maximized. Given a set of data points $D$, such that for $i^{th}$ predictor variable $x_{i}$ and target variable $y_{i}$, $D = \left( {x_{i} ,y_{i} } \right) \forall i \in 1,2,3, \ldots ,N$, where $N$ is the number of sample points, the maximum likelihood estimate of $w$ is given by

1 $$ \tilde{w} = \mathop {\arg \max }\limits_{w} p(D|w) $$

These models concentrate on finding out point estimates which may lead to over-confident decisions for imbalanced classes. To overcome this situation, accounting for uncertainty in the neural networks and estimating weights in the form of probabilistic distributions can lead to a more generalized model that is more robust to imbalanced datasets. Keeping this perspective, BNN was used to predict the various cancer types. According to Bayes theorem, the likelihood $p\left( {w{|}D} \right)$ of observing specific network parameters given the data is expressed as

2 $$ p\left( {w{|}D} \right) = \frac{{p\left( {D{|}w} \right)p\left( w \right)}}{{\int p\left( {D{|}w} \right)p\left( w \right) dx}} $$

where $p(D|w)$ is the probability of occurrence of data given the network parameters, $p\left( w \right)$ is the assumed prior distribution of the network parameters and $p(w|D)$ is the probability of network parameters given the data and is also called posterior distribution.

Tractable solution of $p\left( {w{|}D} \right)$ in case of neural network is computationally not feasible^44^, so a simplified distribution called variational distribution (also called variational posterior, in this case as in Eq. 3) is assumed, which is made to approximate the true posterior by minimizing the KL divergence^45^ between the variational posterior and the true posterior (as in Eq. 4)^40,58^.

3 $$ q\left( {w|\delta } \right) = \mathop \prod \limits_{j} N\left( {w_{j} |\mu_{j} ,\sigma_{j}^{2} } \right) $$

$q\left( {w{|}\delta } \right)$ is the variational posterior of model parameters $w$, $\delta$ is the set of parameters of $q$, $\mu_{j}$ and $\sigma_{j}^{2}$ are the mean and variance of model parameter $w_{j}$.

4 $$ \tilde{\delta } = \mathop {{\text{argmin}}}\limits_{\delta } KL[q(w|\delta )\; ||\; p\;(w|D)] $$

where $KL[A || B]$ denotes KL divergence of B from A, $\tilde{\delta }$ is the estimate of the parameters $\delta$ of the variational distribution $q$.

Since the variational posterior is made to approximate the true posterior, the loss function takes the form

5 $$ L = KL[q\left( {w{|}\delta } \right) || p(w|D)] $$

According to Blundell et al.^41^, using Monte Carlo sampling^59^, the loss function becomes

6 $$ L = \mathop \sum \limits_{i = 1}^{i = n} {\text{log}}\;q(w^{\left[ i \right]} |\delta ) - \log p\left( {w^{\left[ i \right]} } \right) - \log p(D|w^{\left[ i \right]} ) $$

with $i$ denoting the Monte Carlo sample drawn from variational posterior $q(w^{\left[ i \right]} |\delta )$.

BNN used in this study was based on Bayes by Backprop algorithm^41^. The prior was assumed to have a probability distribution $N\left( {0,1} \right)$.

The BNN comprised of three layers. The first layer consisted of 250 neurons, the second layer consisted of 95 neurons and the third layer (output layer) consisted of 31 neurons as there were 31 different types of cancers in our dataset. Sigmoid activation function was used except for the output layer in which Softmax activation function was used. Normal Initialization was used for the weights and Adam’s optimizer was used to update them.

The DNN also comprised of three layers. The first layer consisted of 250, neurons, the second layer consisted of 55 neurons and the output layer consisted of 31 neurons. In DNN too, sigmoid activation before the output layer and Softmax activation in the output layer was used. DNN is l2-regularized. Xavier’s initialization was used to initialize the weights in case this case and Adam optimizer was used to update them.

### Uncertainty estimation and correction

Uncertainty estimation in predictive modelling provides an idea about the confidence of predictions by a model. In a multi-class classification setting, the Softmax activation function in the output layer of the neural network returns the probability value of each class^60^. During inference, Monte Carlo sampling of weights from the approximate variational distribution for $T$ iterations was performed to obtain $T$ predicted probabilities and the following measures of uncertainty as defined by^61^ was used:

7 $$ Epistemic\;Uncertainty,\; \xi = \frac{1}{T}\mathop \sum \limits_{t = 1}^{t = T} \left( {\hat{p}_{t} - \overline{p}} \right)^{T} \left( {\hat{p}_{t} - \overline{p}} \right) $$

where $\hat{p}_{t}$ is the predicted probability by the neural network for *t*th Monte Carlo iteration, $t \in \left[ {1,T} \right]$ and $\overline{p}$ is the mean of the predicted probabilities for $T$ iterations. In our case $\hat{p}_{t}$ is a $c \times 1$ dimensional vector, c being the number of classes.

In the matrix representing Epistemic Uncertainty (from Eq. 7), the diagonal elements were considered for our analysis as these elements involved calculations related to a single probability value and represented the variance of the predicted output. Among the diagonal elements, the element that corresponded to the class predicted by the model were considered. In this case, the equation boiled down to:

8 $$ Epistemic \;Uncertainty,\; \xi_{i} = \frac{1}{T}\mathop \sum \limits_{t = 1}^{t = T} (\hat{p}_{t}^{i} - \overline{p}_{t}^{i} )^{2} $$

where $i$ was the index of the predicted class. This gave us the uncertainty estimate of the class predicted by the model.

After calculating the uncertainty values, the predictive outputs were corrected for the uncertainty associated with them. For each cancer type, a linear model between the log odds ratio of the expected value of the predicted output from $T$ Monte Carlo iterations and the square root of epistemic uncertainty $\xi_{i}$ was assumed. Ordinary Least Squares (OLS) was then used to calculate the coefficients $\alpha$ and $\beta$

$$ f\left( {E\left[ {\hat{p}_{i} } \right]} \right) = \alpha + \beta \surd \xi_{i} + \varepsilon $$

where the model error was assumed by $\varepsilon \sim N\left( {0,\sigma^{2} } \right)$.

The function $f$ can be defined as:

$$ f\left( x \right) = ln\left( {\frac{x}{1 - x}} \right) $$

After estimating the coefficients, the corrected probability values $p_{corr}$ were obtained as

$$ \widehat{{p_{corr, i} }} = f^{ - 1} \left( {E\left[ {\widehat{{p_{i} }}} \right] - \beta \xi_{i} } \right) $$

### Evaluation metrics

For evaluating the performance of the classifiers, the following evaluation metrics were used:

$$ Overall\;Accuracy = \frac{Number\;of\;correct\;predictions}{{Total\;number\;of\;predictions}} = \frac{TP + FN}{{TP + FN + FP + TN}} $$

$$ Precision\;\left( P \right) = \frac{TP}{{TP + FP}} $$

$$ Recall\;\left( R \right) = \frac{TP}{{TP + FN}} $$

$$ F1\;Score\; = \frac{2 \times P \times R}{{P + R}} $$

where $TP$ denotes True Positive, $FP$ denotes false positives and $FN$ denotes false negatives.

### Single-gene classification of cancer types

Whether a small subset of 103 feature genes identified by PCA could classify specific cancer types was also tested. To do so, performance of each individual gene in cancer type classification was evaluated (Supplementary fig. S3). Several genes had both Precision and Recall values greater than 0.75 (Supplementary fig. S4A) for certain cancer types. These included six genes (*MYO1F, COL16A1, ANTXR1, TMEM54, PCGF2* and *PARVA)* for Acute Myeloid Leukaemia (LAML) and one gene (*TARP)* was selected for Prostate Adenocarcinoma (PRAD). These genes had unique and distinctive expression signature in the corresponding cancer types and thus, were able to classify them accurately (Supplementary fig. S4B). This analysis was further extended to more than one gene by selecting top ranked 20 genes according to their F1 scores. Interestingly, 10 cancer types could be classified with just 12 genes with Precision and Recall values greater than 0.75 (Supplementary fig. S4C; Supplementary table S4). These included Acute Myeloid Leukaemia (LAML,1 gene), Prostate Adenocarcinoma (PRAD,1 gene), Thyroid Carcinoma (THCA,2 genes), Lower Grade Glioma (LGG,2 genes), Kidney Renal Clear Cell Carcinoma (KIRC,3 genes), Liver Hepatocellular Carcinoma (LIHC,3 genes), Breast Invasive Carcinoma (BRCA,5 genes), Kidney Renal Papillary Cell Carcinoma (KIRP,6 genes), Stomach Adenocarcinoma (STAD,11 genes), Skin Cutaneous Melanoma (SKCM,12 genes) (Supplementary fig. S4C).

## Supplementary Information

Supplementary Legends.

Supplementary Information 2.

## Data Availability

The datasets analysed are publicly available from UCSC Xena (http://xena.ucsc.edu/) and ICGC (https://dcc.icgc.org). https://github.com/pjoshi-hub/Bayesian_classification_model.
