# Supplementary material for: EpICC: A Bayesian neural network model with uncertainty correction for a more accurate classification of cancer
Source: Sci Rep. 2022 Aug 26;12:14628. doi: 10.1038/s41598-022-18874-6 (PMC9418241; doi:10.1038/s41598-022-18874-6)
Supplement: Supplementary file 1 — Supplementary Legends. [file 41598_2022_18874_MOESM1_ESM.docx]

**Supplementary figures**

**Figure S1 - Feature selection by two step principal component analysis (PCA) and logistic regression (a)** Scatterplot obtained after 1^st^ Principal Components Analysis on the gene expression data. The x-axis denotes the 1^st^ Principal Component and the y-axis denotes the 2^nd^ Principal Component. The plotted data points represent individual samples of the cancer types mentioned in the legends. **(b)** Scatterplot obtained after 2^nd^ Principal Components Analysis. The x-axis denotes the 1^st^ Principal Component and the y-axis denotes the 2^nd^ Principal Component. The plotted data points represent individual samples of different cancer types. **(c)** Plot for selecting the values of $m$ and $n$ in case of feature selection by the combination of PCA and Logistic Regression, with $m$ and $n$ denoting the number of features selected in 1^st^ and 2^nd^ Principal Components Analysis respectively. Five-fold cross-validation accuracies are plotted for each $m$ and $n$. **(d)** Plot showing the increase in percentage variance explained after selecting the same number of markers by two PCA steps as compared to just one step.

**Figure S2 – Expression level of 103 marker genes selected by PCA and logistic regression across 31 cancer types.**

**Figure S3 - Precision, Recall and F1 Score of 103 marker genes in individual gene wise classification.** Individual genes were used to classify al the 31 cancer types using L2-regularized Logistic Regression and the precision, recall and F1 score for each classification are shown in the heatmaps. The horizontal axis shows the cancer types and the vertical axis shows individual genes.

**Figure S4 -** **A small number of genes can classify up to 10 cancer types. (a)** Precision, Recall and F1 Scores for the genes which showed both precision and recall greater than 0.75 in single gene classification method. The x-axis shows the genes and the cancer types for which the genes exhibited high precision and recall values. **(b)** Boxplots of the expression values of the genes that show both precision and recall values greater than 0.75 in single gene classification method. The expression levels of these genes were compared between the cancer types that they classified with a high precision and recall and the rest of the cancer types. **(c)** Plot showing the number of cancer types (along with the name of the cancer types) that are classified (y-axis) by a small number of genes up to 20 genes (x-axis). For each cancer type, the genes were sorted in descending order according to F1 scores obtained from single gene classification results and then a particular number of genes (the value in the x-axis) having the highest F1 scores were selected. These selected genes were used for classification.

**Figure S5** – **Distribution of epistemic uncertainty in classification of cancer types.** The plots show distribution of epistemic uncertainty in training data (green), in test data that were correctly classified (blue) and in tests data that were incorrectly classified (red). The uncertainty was substantially higher in test data that were incorrectly classified compared to that in the test data that were correctly classified.

**Figure S6 – Comparison of model-based uncertainty values of the correct and incorrect predictions.** The green bars represent the mean uncertainty of correct predictions on training data, the blue bars show the mean uncertainty of correct predictions on test data and the red bars show the mean uncertainty of incorrect predictions on the test data for (**a)** LGG subtypes **(b)** BRCA subtypes **(c)** ESCA subtypes **(d)** THCA subtypes.

**Figure S7 -** **Plots showing changes in overall accuracy and the percentage of sample retained for prediction with changes in the filtering cut-off.** The plots show data for prediction of LGG, BRCA, ESCA and THCA subtypes. The x-axis represents the filtering cut-off as multiples of the uncertainty value of correct predictions in training. The y-axes represent the overall accuracy and the percentage of samples whose uncertainty is lower than the filtering cut-off.
