# Supplementary material for: EpICC: A Bayesian neural network model with uncertainty correction for a more accurate classification of cancer
Source: Sci Rep. 2022 Aug 26;12:14628. doi: 10.1038/s41598-022-18874-6 (PMC9418241; doi:10.1038/s41598-022-18874-6)
Supplement: Supplementary file 2 — Supplementary Information 2. [file 41598_2022_18874_MOESM2_ESM.pdf]

Supplementary material

EpICC: A Bayesian Neural Network model with uncertainty correction for a more accurate classification of cancer

Prasoon Joshi, Riddhiman Dhar

This PDF file contains

Figures S1 to S7

Tables S1 to S4

Figure S1

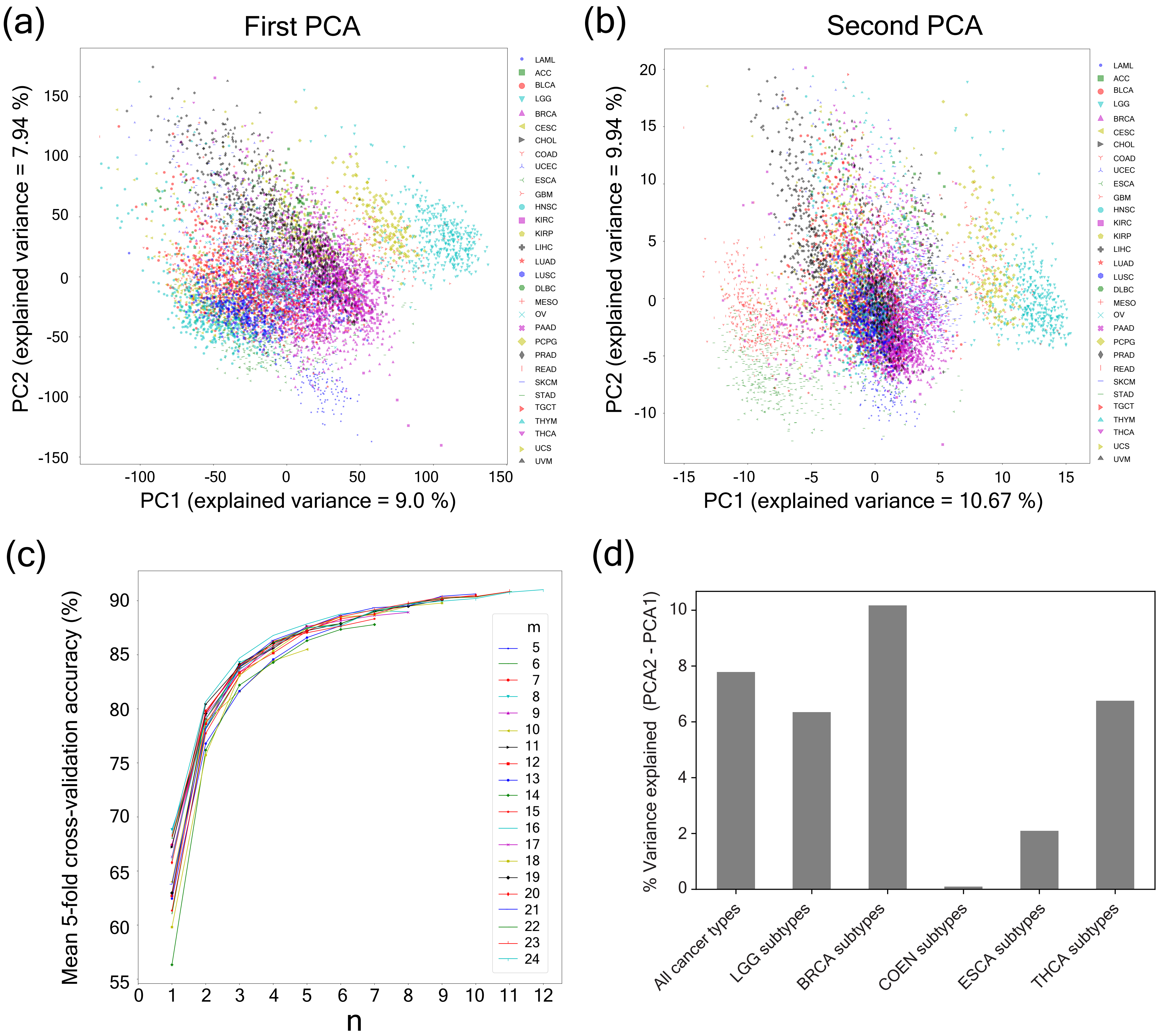

(a)
First PCA
PC1 (explained variance = 9.0 %)
PC2 (explained variance = 7.94 %)
LAML
ACC
BLCA
LGG
BRCA
CESC
CHOL
COAD
UCEC
ESCA
GBM
HNSC
KIRC
KIRP
LIHC
LUAD
LUSC
DLBC
MESO
OV
PAAD
PCPG
PRAD
READ
SKCM
STAD
TGCT
THYM
THCA
UCS
UVM
(b)
Second PCA
PC1 (explained variance = 10.67 %)
PC2 (explained variance = 9.94 %)
(c)
Mean 5-fold cross-validation accuracy (%)
n
m
(d)
% Variance explained (PCA2 - PCA1)
All cancer types
LGG subtypes
BRCA subtypes
COEN subtypes
ESCA subtypes
THCA subtypes

Figure S2

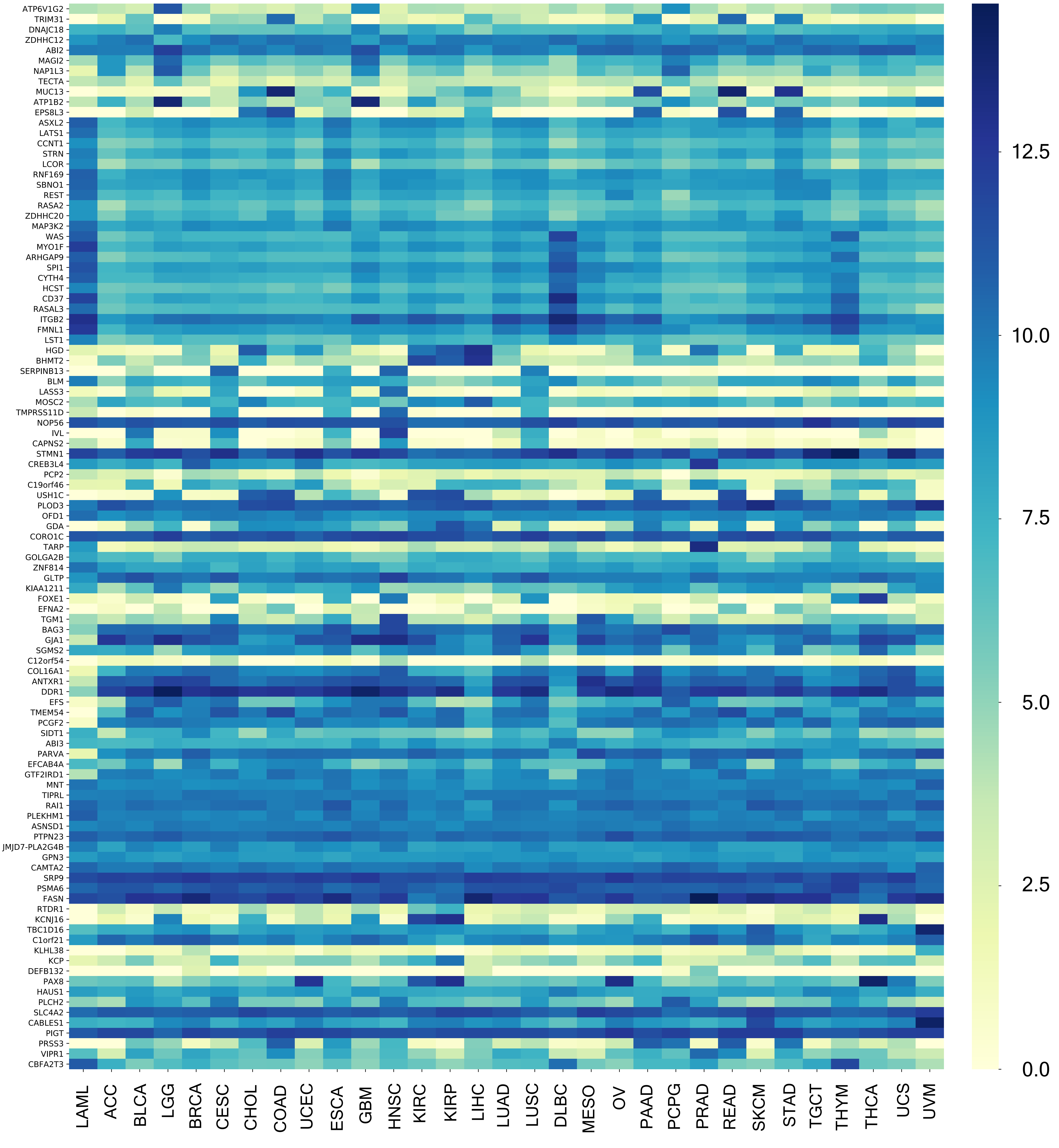
ATP6V1G2
TRIM31
DNAJC18
ZDHHC12
ABI2
MAGI2
NAP1L3
TECTA
MUC13
ATP1B2
EPS8L3
ASXL2
LATS1
CCNT1
STRN
LCOR
RNF169
SBNO1
REST
RASA2
ZDHHC20
MAP3K2
WAS
MYO1F
ARHGAP9
SPI1
CYTH4
HCST
CD37
RASAL3
ITGB2
FMNL1
LST1
HGD
BHMT2
SERPINB13
BLM
LASS3
MOSC2
TMPRSS11D
NOP56
IVL
CAPNS2
STMN1
CREB3L4
PCP2
C19orf46
USH1C
PLOD3
OFD1
GDA
CORO1C
TARP
GOLGA2B
ZNF814
GLTP
KIAA1211
FOXE1
EFNA2
TGM1
BAG3
GJA1
SGMS2
C12orf54
COL16A1
ANTXR1
DDR1
EFS
TMEM54
PCGF2
SIDT1
ABI3
PARVA
EFCAB4A
GTF2IRD1
MNT
TIPRL
RAI1
PLEKHM1
ASNSD1
PTPN23
JMJD7-PLA2G4B
GPN3
CAMTA2
SRP9
PSMA6
FASN
RTDR1
KCNJ16
TBC1D16
C1orf21
KLHL38
KCP
DEFB132
PAX8
HAUS1
PLCH2
SLC4A2
CABLES1
PIGT
PRSS3
VIPR1
CBFA2T3
LAML
ACC
BLCA
LGG
BRCA
CESC
CHOL
COAD
UCEC
ESCA
GBM
HNSC
KIRC
KIRP
LIHC
LUAD
LUSC
DLBC
MESO
OV
PAAD
PCPG
PRAD
READ
SKCM
STAD
TGCT
THYM
THCA
UCS
UVM
12.5
10.0
7.5
5.0
2.5
0.0

Figure S3

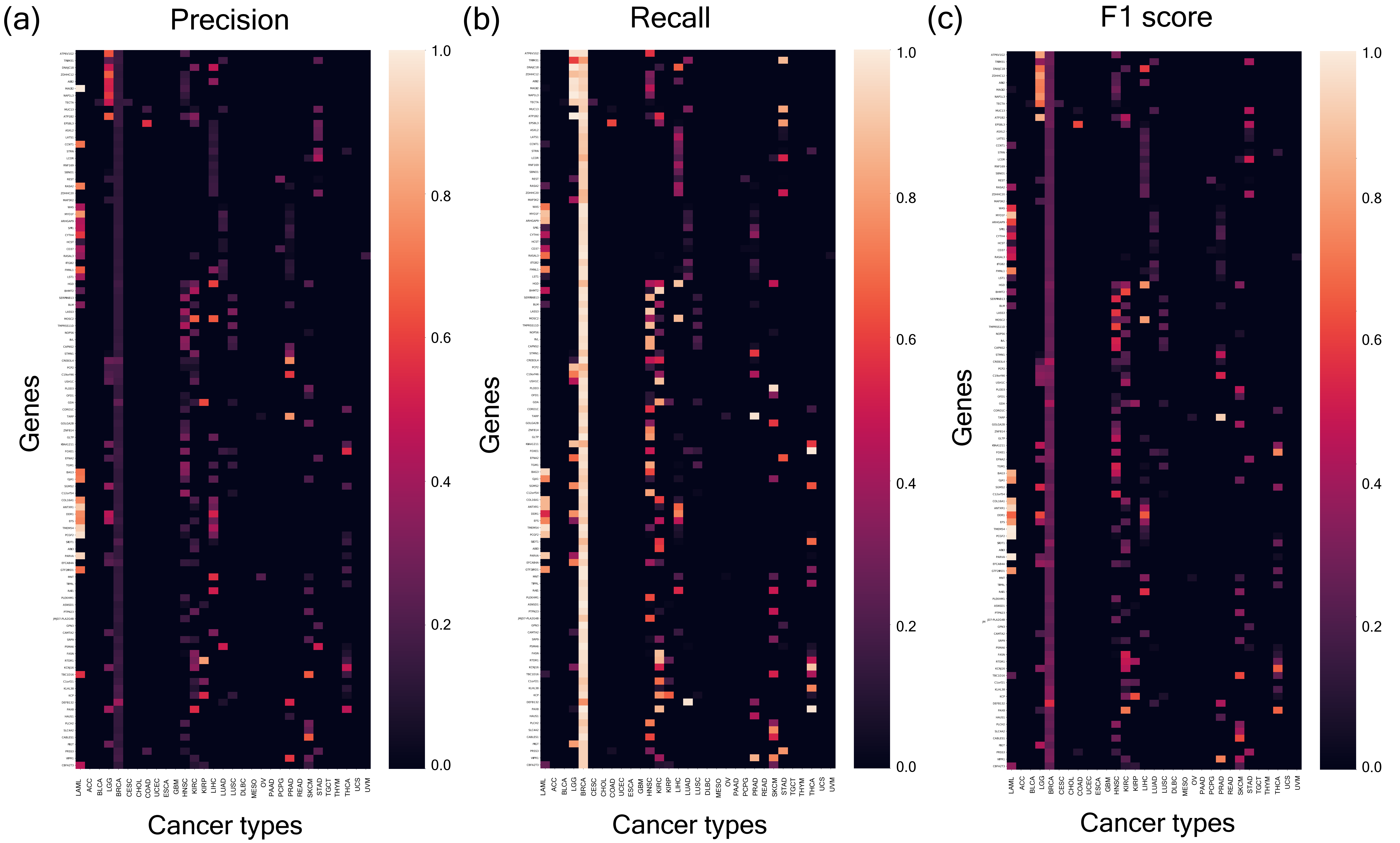
(a)
Precision
(b)
Recall
(c)
F1 score
Genes
Cancer types
1.0
0.8
0.6
0.4
0.2
0.0
LAML ACC BLCA LGG BRCA CESC CHOL COAD UCEC ESCA GBM HNSC KIRC KIRP LIHC LUAD LUSC DLBC MESO OV PAAD PCPG PRAD READ SKCM STAD TGCT THYM THCA UCS UVM

Figure S4

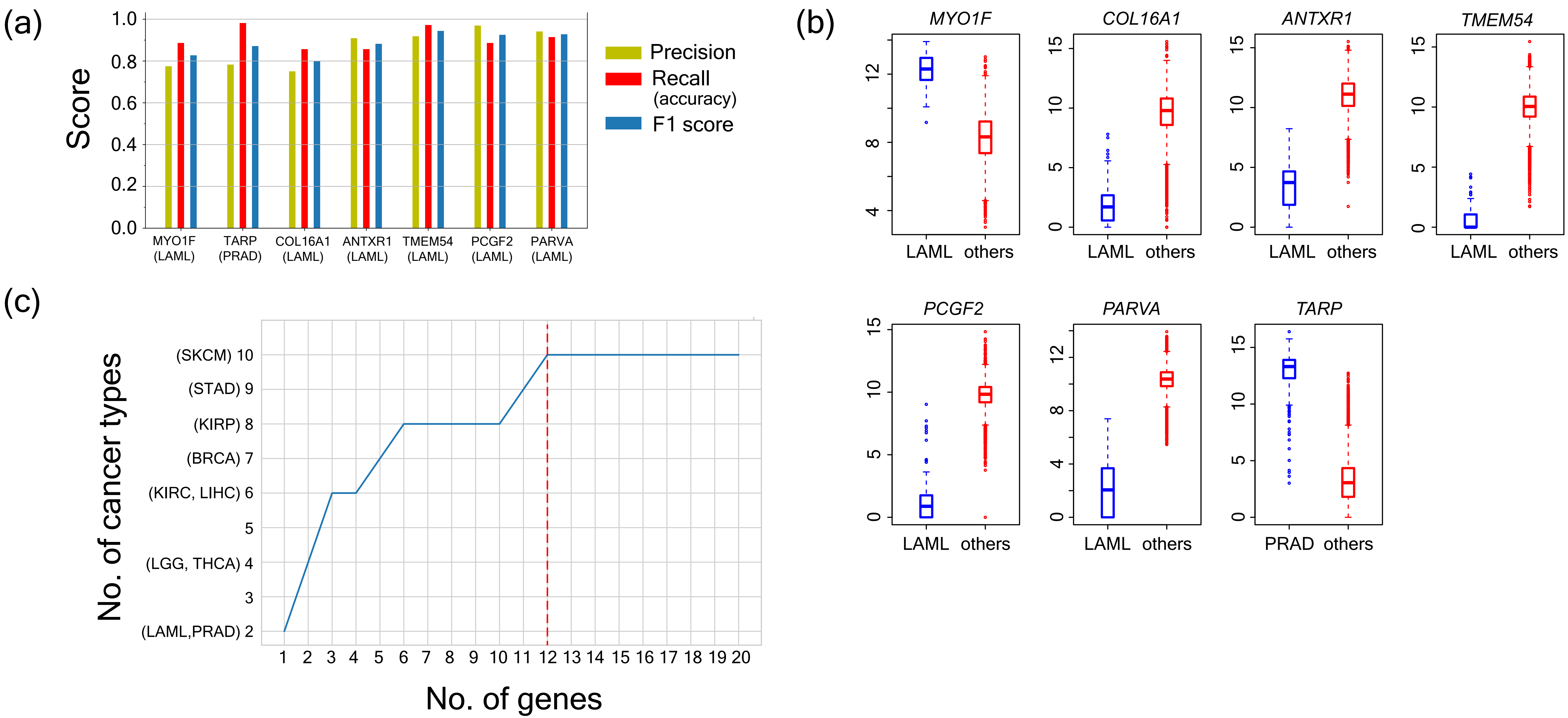

(a)
Score
1.0
0.8
0.6
0.4
0.2
0.0
MYO1F (LAML)
TARP (PRAD)
COL16A1 (LAML)
ANTXR1 (LAML)
TMEM54 (LAML)
PCGF2 (LAML)
PARVA (LAML)
Precision
Recall (accuracy)
F1 score
(b)
MYO1F
COL16A1
ANTXR1
TMEM54
PCGF2
PARVA
TARP
LAML
others
PRAD
(c)
No. of cancer types
(SKCM) 10
(STAD) 9
(KIRP) 8
(BRCA) 7
(KIRC, LIHC) 6
5
(LGG, THCA) 4
3
(LAML,PRAD) 2
1 2 3 4 5 6 7 8 9 10 11 12 13 14 15 16 17 18 19 20
No. of genes

Figure S5

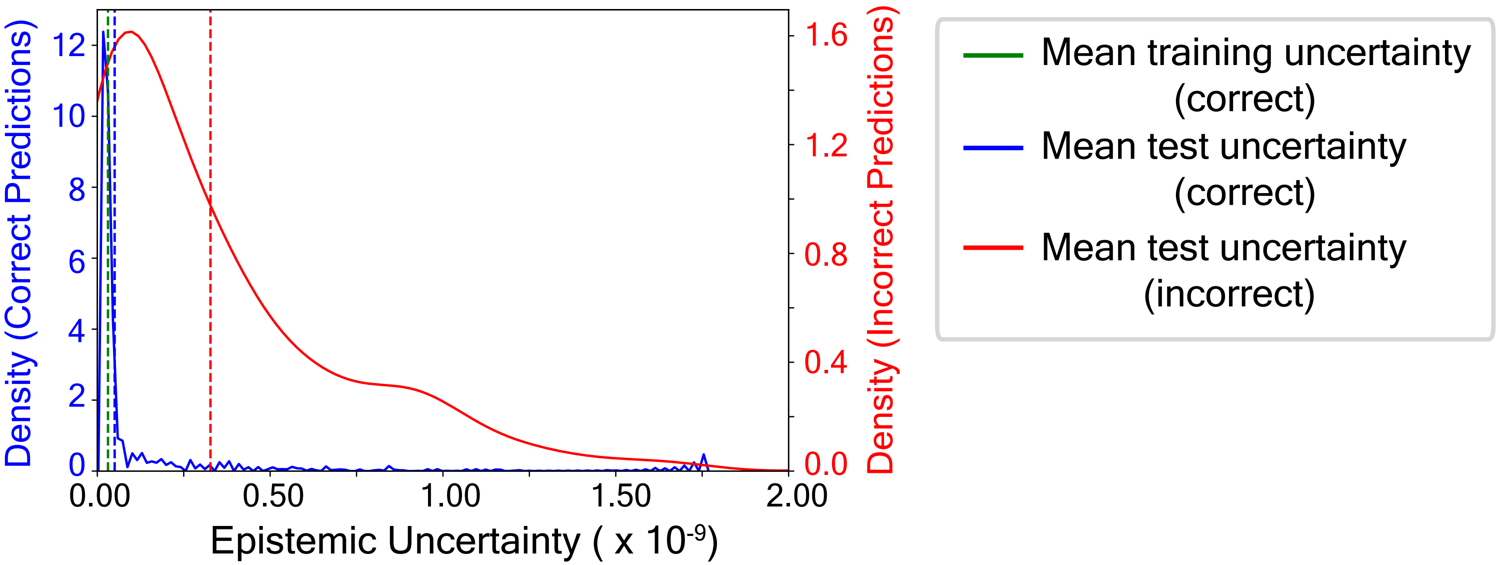

Density (Correct Predictions)
0
2
4
6
8
10
12
Density (Incorrect Predictions)
0.0
0.4
0.8
1.2
1.6
0.00
0.50
1.00
1.50
2.00
Epistemic Uncertainty (x 10-9)
Mean training uncertainty (correct)
Mean test uncertainty (correct)
Mean test uncertainty (incorrect)

Figure S6

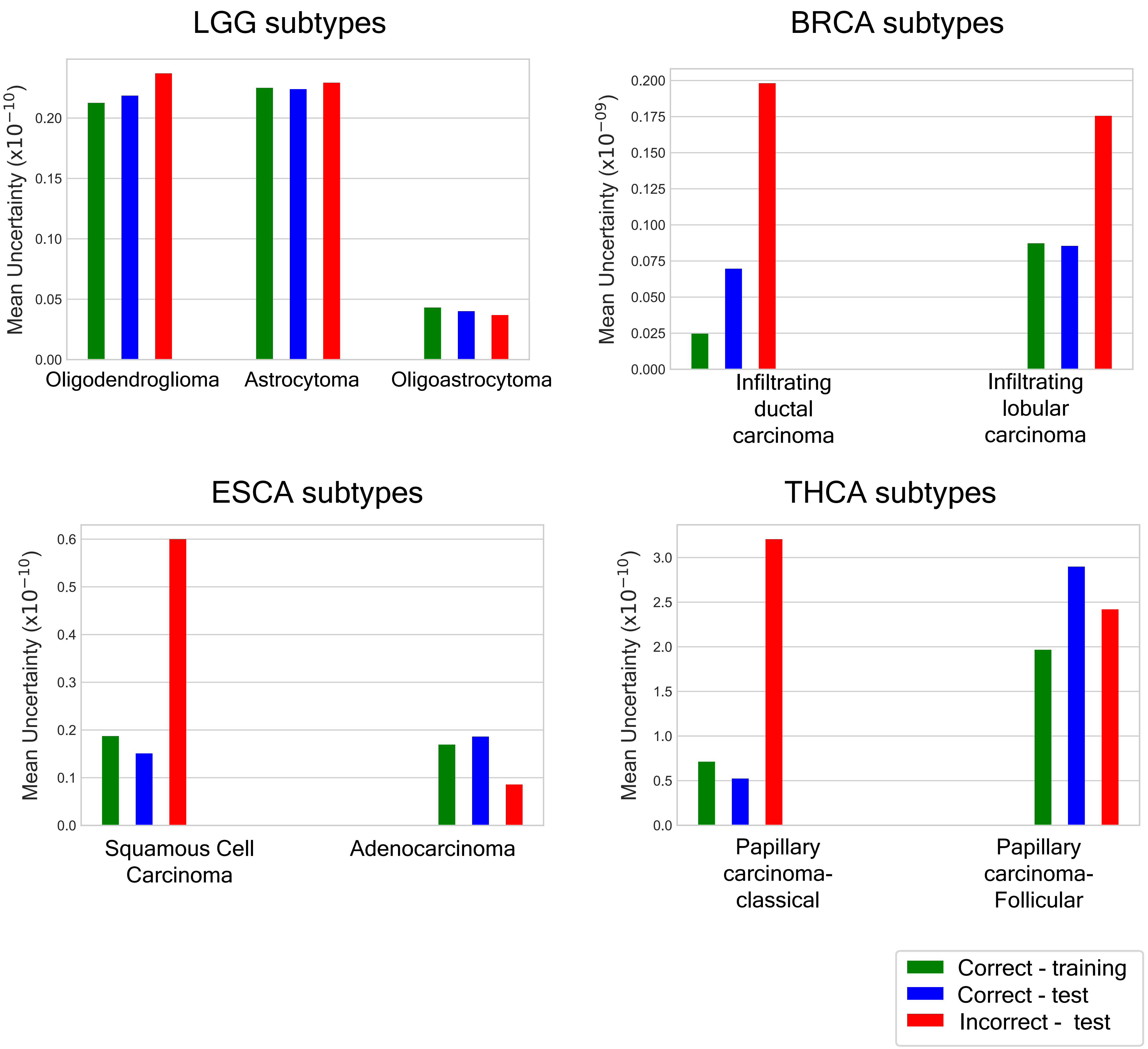
LGG subtypes
Mean Uncertainty (x10^{-10})
0.00
0.05
0.10
0.15
0.20
Oligodendroglioma
Astrocytoma
Oligoastrocytoma
BRCA subtypes
Mean Uncertainty (x10^{-09})
0.000
0.025
0.050
0.075
0.100
0.125
0.150
0.175
0.200
Infiltrating ductal carcinoma
Infiltrating lobular carcinoma
ESCA subtypes
Mean Uncertainty (x10^{-10})
0.0
0.1
0.2
0.3
0.4
0.5
0.6
Squamous Cell Carcinoma
Adenocarcinoma
THCA subtypes
Mean Uncertainty (x10^{-10})
0.0
0.5
1.0
1.5
2.0
2.5
3.0
Papillary carcinoma-classical
Papillary carcinoma-Follicular
Correct - training
Correct - test
Incorrect - test

Figure S7

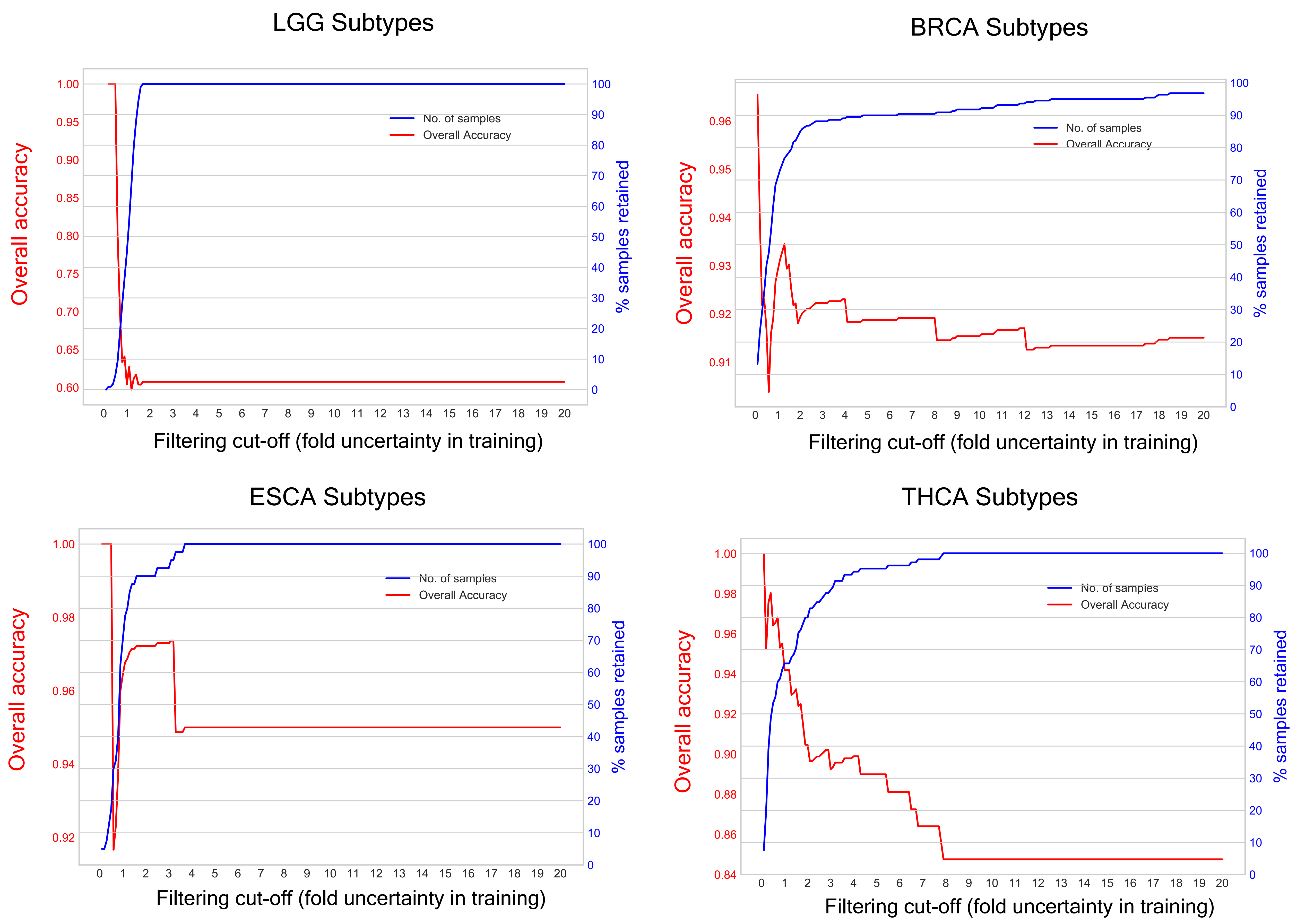

LGG Subtypes
No. of samples
Overall Accuracy
Overall accuracy
% samples retained
Filtering cut-off (fold uncertainty in training)
BRCA Subtypes
No. of samples
Overall Accuracy
Overall accuracy
% samples retained
Filtering cut-off (fold uncertainty in training)
ESCA Subtypes
No. of samples
Overall Accuracy
Overall accuracy
% samples retained
Filtering cut-off (fold uncertainty in training)
THCA Subtypes
No. of samples
Overall Accuracy
Overall accuracy
% samples retained
Filtering cut-off (fold uncertainty in training)

Table S1 - Details of 103 feature genes

S. No.	Gene	Description	Function	Reported Oncogenic? (Y/N)	Reported tumor suppressor? (Y/N)	Biomarker/Other association with cancer/? (Y/N)
1	ABI2	Abl interactor 2	Actin polymerization/depolymerization, cell migration, cytoskeleton organization	N	Y (Dai and Pendergast, 1995)	N
2	ABI3	ABI family member 3	Regulation of actin polymerization, cell migration	N	Y (Latini *et al.* , 2011; Moraes *et al* ., 2016)	N
3	ANTXR1	ANTXR cell adhesion molecule 1	Role in cell attachment and migration, interacts with extracellular matrix proteins and with the actin cytoskeleton.	N	N	Y (Chen *et al* ., 2013; Geng *et al.* , 2019)
4	ARHGAP9	Rho GTPase activating protein 9	GTPase activator for the Rho-type GTPases	N	Y (Piao *et al.* , 2020)	Y (Piao *et al.* , 2020)
5	ASNSD1	Asparagine synthetase domain containing 1	Asparagine synthetic pathway	N	N	N
6	ASXL2	ASXL transcriptional regulator 2	Epigenetic regulators that bind various histone-modifying enzymes and involved in the assembly of transcription factors. Mutations in this gene are associated with cancer in several tissues (breast, bladder, pancreas, ovary, prostate, and blood)	Putative (Park et al., 2016; Cui et al., 2020)	Y(Daou et al., 2015; Micol et al., 2017)	Y (Park et al., 2016; Cui et al., 2020)
7	ATP1B2	ATPase Na+/K+ Transporting Subunit Beta 2	Non-catalytic component of Na(+)-K(+)-ATPase	Putative (Li et al., 2019)	Putative (van den Boome et al., 2006; Demircan et al., 2009)	N
8	ATP6V1G2	ATPase H+ Transporting V1 Subunit G2	Component of vacuolar ATPase (V-ATPase) that is responsible for acidification of intracellular compartment	N	N	Y (Sennoune et al., 2004; Xu et al., 2013; Terrasi et al., 2019)
9	BAG3	BAG Cochaperone 3	Co-chaperone for HSP70 and HSC70 chaperone proteins, has anti-apoptotic activity	Y (Chiappetta et al., 207; Shi *et al.* , 2018; Li et al., 2018)	Y (Kong et al., 2016)	N
10	BHMT2	Betaine--Homocysteine S-Methyltransferase 2	Regulation of homocysteine metabolism, converts homocysteine to methionine using S-methylmethionine (SMM) as a methyl donor	N	N	Putative (Dufresne et al., 2019)
11	BLM	Bloom Syndrome RecQ Like Helicase	TP-dependent DNA helicase that unwinds single- and double-stranded DNA, participates in DNA replication and repair	N	Y (Payne and Hickson, 2009; de Voer et al., 2015)	Y (Arora et al., 2015)
12	C12orf54	Chromosome 12 Open Reading Frame 54	-	N	N	N
13	C19orf46	SYNE4 (Spectrin Repeat Containing Nuclear Envelope Family Member 4)	component of the LINC (LInker of Nucleoskeleton and Cytoskeleton) complex, involved in the connection between the nuclear lamina and the cytoskeleton	N	N	Y (Xu et al., 2020)
14	C1orf21	Chromosome 1 Open Reading Frame 21	Cell Proliferation-Inducing Gene 13	N	N	Y (Hannemann et al., 2006; Lee et al., 2007; Yoshihara et al., 2015)
15	CABLES1	CDK5 And ABL1 Enzyme Substrate 1	Cyclin-dependent kinase binding protein. Enhances cyclin-dependent kinase tyrosine phosphorylation by nonreceptor tyrosine kinases, such as that of CDK5 by activated ABL1	N	Y (Arnason et al., 2013; Shi et al., 2015)	Putative (Huang et al., 2017)
16	CAMTA2	Calmodulin-Binding Transcription Activator 2	Calmodulin-binding transcription activator protein	Putative (Luan et al., 2019)	N	N
17	CAPNS2	Calcium-Dependent Protease Small Subunit 2	Calcium-regulated non-lysosomal thiol-protease	N	N	N
18	CBFA2T3	Core-Binding Factor, Runt Domain, Alpha Subunit 2; Translocated To, 3	Transcriptional corepressor, facilitates transcriptional repression via association with DNA-binding transcription factors and recruitment of other corepressors and histone-modifying enzymes	Y (Masetti *et al.* , 2019)	Y (Kochetkova et al., 2002)	N
19	CCNT1	Cyclin T1	Regulatory subunit of the cyclin-dependent kinase pair (CDK9/cyclin-T1) complex	Y (Moiola et al., 2010)	N	N
20	CD37	CD37 Molecule	Leukocyte antigen CD37	N	Y (de Winde et al., 2016; Schaper and van Spriel, 2018)	Y (Zhang et al., 2020)
21	COL16A1	Collagen, Type XVI, Alpha 1	Involved in cell attachment and cell spreading	Y (Senner, 2008; Bauer, 2011; Ratzinger, 2011)	N	Y (Yoshihara et al., 2015)
22	CORO1C	Coronin, Actin-Binding Protein, 1C	Role in directed cell migration by regulation of RAC1	Y (Castagnino et al., 2018; Cheng et al., 2019)	N	Y (Wu et al., 2010; Cheng et al., 2019; Wang et al., 2020)
23	CREB3L4	Cyclic AMP-Responsive Element-Binding Protein 3-Like Protein 4	Transcriptional activator that may play a role in the unfolded protein response	Y (Chen et al., 2010; Kim et al., 2017)	N	Y (Kim et al., 2017)
24	CYTH4	Cytohesin 4	Promotes the activation of ARF factors	N	N	N
25	DDR1	Discoidin Domain Receptor Tyrosine Kinase 1	Tyrosine kinase that functions as cell surface receptor for fibrillar collagen and regulates cell attachment to the extracellular matrix, remodeling of the extracellular matrix	Y (Valiathan et al., 2012; Hu et al, 2014)	Y (Takai et al., 2018)	Y (Song et al., 2016)
26	DEFB132	Defensin Beta 132	Cysteine-rich cationic polypeptides that are important in the immunologic response to invading microorganisms	N	N	N
27	DNAJC18	DnaJ Heat Shock Protein Family (Hsp40) Member C18	DNAJC18 (DnaJ Heat Shock Protein Family (Hsp40) Member C18)	N	N	Y (Guey et al., 2010; Pohl et al., 2019)
28	EFCAB4A	EF-hand calcium binding domain 4A	calcium ion binding	N	N	Y (Milani et al., 2010)

29	EFNA2	Ephrin A2	Cell surface GPI-bound ligand for Eph receptors, a family of receptor tyrosine kinases crucial for migration, repulsion and adhesion during neuronal, vascular and epithelial development	N	N	N
30	EFS	Embryonal Fyn-Associated Substrate	Central coordinating role for tyrosine-kinase-based signaling related to cell adhesion	N	Y (Sertkaya et al., 2015)	Y (Neumann et al., 2011)
31	EPS8L3	Epidermal Growth Factor Receptor Pathway Substrate 8-Related	Epidermal growth factor receptor pathway substrate 8 (EPS8), a substrate for the epidermal growth factor receptor.	Y (Zeng et al., 2018; Li et al., 2019; Chen et al., 2020; Xuan et al., 2020)	N	Y (Li et al., 2019)
32	FASN	Fatty Acid Synthase	Catalyze the synthesis of palmitate from acetyl-CoA and malonyl-CoA, in the presence of NADPH, into long-chain saturated fatty acids	Y (Patel et al., 2015; Corominas-Faja et al., 2017)	N	Y (Visca et al., 2004; Cruz et al., 2014; Ricklefs et al., 2020)
33	FMNL1	Formin Like 1	Formin-related proteins have been implicated in morphogenesis	Putative (Chen et al., 2018)	N	Y (Chen et al., 2018; Higa et al., 2019)
34	FOXE1	Forkhead Box E1	Forkhead family of transcription factors	Putative (Ma et al., 2019)	Y (Dai et al., 2020)	Y (Kallel et al., 2010; Papadia et al., 2014; Sugimachi et al., 2016)
35	GDA	Guanine Deaminase	Enzyme responsible for the hydrolytic deamination of guanine	N	N	N
36	GJA1	Gap Junction Protein Alpha 1	Gap junction protein - a gap junction consists of a cluster of pairs of transmembrane channels, the connexons, through which low MW materials diffuse	N	Y(Talhouk et al., 2013; Teleki et al., 2014; Naser Al Deen et al., 2019)	Putative (Aasen et al., 2019)
37	GLTP	Glycolipid Transfer Protein	Involved in intermembrane transfer of glycolipids	N	Y (Mishra et al., 2019)	N
38	GOLGA2B	Golgin A2 Pseudogene 5	Pseudogene	N	N	N
39	GPN3	GPN-Loop GTPase 3	Small GTPase required for proper localization of RNA polymerase II	Putative (Lara-Chacón et al., 2019)	N	N
40	GTF2IRD1	GTF2I Repeat Domain Containing 1	Transcription regulator involved in cell-cycle progression and skeletal muscle differentiation	Y (Huo et al., 2016; Nambara et al., 2020)	N	N
41	HAUS1	HAUS Augmin Like Complex Subunit 1	Mitotic spindle assembly, maintenance of centrosome integrity and completion of cytokinesis as part of the HAUS augmin-like complex	N	N	Y (Campregher et al., 2016)
42	HCST	Hematopoietic Cell Signal Transducer	Transmembrane adapter protein forms an activation receptor KLRK1-HCST; KLRK1-HCST receptor plays a role in immune surveillance against tumors and is required for cytolysis of tumors cells	N	N	N
43	HGD	Homogentisate 1,2-Dioxygenase	Involved in the catabolism of the amino acids tyrosine and phenylalanine	N	N	N
44	ITGB2	Integrin Subunit Beta 2	participate in cell adhesion as well as cell-surface mediated signalling	N	N	Y (Laskowska et al., 2016; Peng et al., 2020)
45	IVL	Involucrin	Part of the insoluble cornified cell envelope (CE) of stratified squamous epithelia	N	N	Y (Walts et al., 1985)
46	JMJD7-PLA2G4B	Jumonji Domain Containing 7-Phospholipase A2, Group IVB (Cytosolic) Read-Through	-	Y (Cheng et al., 2017)	N	N
47	KCNJ16	Potassium Channel, Inwardly Rectifying Subfamily J Member 16	An integral membrane protein and inward-rectifier type potassium channel	N	N	Y (Adam et al., 2010; Jiang et al., 2017)
48	KCP	Kielin/Chordin-Like Protein	Enhances bone morphogenetic protein (BMP) signaling	N	N	N
49	KIAA1211	-	Uncharacterized	Y (Liu et al., 2019)	N	Y (Zhou et al., 2019)
50	KLHL38	Kelch Like Family Member 38	-	N	N	Y (Schleifer et al., 2018; Kuang et al., 2020)
51	LASS3	Ceramide Synthase 3	Regulate sphingolipid synthesis by catalyzing the formation of ceramides from sphingoid base and acyl-coA substrates	N	N	Y (Dany et al., 2015; Moro et al., 2018; Brachtendorf et al., 2019)
52	LATS1	Large Tumor Suppressor Kinase 1	Serine/threonine-protein kinase	N	Y (Xia et al., 2002; Yang et al., 2004; Hao et al., 2008)	Y (Ji et al., 2012; Furth et al., 2018)
53	LCOR	Ligand Dependent Nuclear Receptor Corepressor	Repressor of ligand-dependent transcription activation	N	N	Y(Jalaguier et al., 2017; Triki et al., 2017; Vogelsang et al., 2020)
54	LST1	Leukocyte Specific Transcript 1	A membrane protein that can inhibit the proliferation of lymphocytes, possible role in immunmodulation	N	N	Y (Hsu et al., 2019)
55	MAGI2	Membrane Associated Guanylate Kinase, WW And PDZ Domain	Act as scaffold molecule at synaptic junctions by assembling neurotransmitter receptors and cell adhesion proteins	Putative (Li et al., 2020)	Y (Thomas et al., 2002; Hu et al., 2007; Berger et al., 2011; Ma et al., 2016)	Y (Mahdian et al., 2014; Valle et al., 2020)

56	MAP3K2	Mitogen-Activated Protein Kinase Kinase Kinase 2	Component of protein kinase signal transduction cascade. Mediates activation of the NF-kappa-B, AP1 and DDIT3 transcriptional regulators	Y (Mazur et al., 2014; Huang et al., 2016; Wang et al., 2018; Shi et al., 2020)	N	N
57	MNT	MAX Network Transcriptional Repressor	Binds DNA as a heterodimer with MAX and represses transcription.	Y (Link et al., 2012; Link and Hurlin, 2015)	N	N
58	MOSC2	Mitochondrial Amidoxime Reducing Component 2 (MARC2)	Catalyzes the reduction of N-oxygenated molecules, acting as a counterpart of cytochrome P450 and flavin-containing monooxygenases in metabolic cycles	Y (Wu et al., 2020)	N	Y (Wu et al., 2020)
59	MUC13	Mucin 13, Cell Surface Associated	Epithelial and hemopoietic transmembrane mucin that may play a role in cell signaling.	Y (Sheng et al., 2017; Khan et al., 2017; Kumari et al., 2018; Sheng et al., 2019; Tiemin et al., 2020)	N	Y (Khan et al., 2018; Filippou et al., 2018)
60	MYO1F	Unconventional myosin type 1F	Use energy from ATP hydrolysis to generate force on actin filaments, works in intracellular environment	Y (Diquigiovanni e tal., 2018)	N	Y (Taki et al., 2005)
61	NAP1L3	Nucleosome Assembly Protein 1 Like 3	A member of the nucleosome assembly protein (NAP) family	N	N	Y (Ross et al., 2015)
62	NOP56	NOP56 Ribonucleoprotein	Involved in 60S ribosomal subunit biogenesis. Core component of snoRNP particles	Y (Cowling et al., 2014)	N	Y (Gong et al., 2017; Toth et al., 2019)
63	OFD1	OFD1 Centriole And Centriolar Satellite Protein	omponent of the centrioles controlling mother and daughter centrioles length	N	N	N
64	PARVA	Parvin Alpha	Plays a role in sarcomere organization and in smooth muscle cell contraction	Y (Fukuda et al., 2013; Huang et al., 2015; Velazquez-Torres et al., 2018)	N	N
65	PAX8	Paired Box 8	Involved in thyroid follicular cell development and expression of thyroid-specific genes	Y (Kroll et al., 2000; Allison, 2019)	N	Y (Nonaka et al., 2008; Wang et al., 2013; Xiang and Kong, 2013; Wang et al., 2015)
66	PCGF2	Polycomb Group Ring Finger 2	Transcriptional repressor, May play a role in control of cell proliferation and/or neural cell development.	N	Y (Guo et al., 2007; Lee et al., 2014)	Y (Zakrzewska et al., 2011)
67	PCP2	Purkinje Cell Protein 2	May function as a cell-type specific modulator for G protein-mediated cell signaling	N	Putative (Yan et al., 2006)	N
68	PIGT	Phosphatidylinositol Glycan Anchor Biosynthesis Class T	Involved in glycosylphosphatidylinositol (GPI)-anchor biosynthesis	Y (Wu et al., 2002; Nagpal et al., 2008)	N	Y (Wu et al., 2002; Nagpal et al., 2008)
69	PLCH2	Phospholipase C Eta 2	Member of the PLC-eta family of the phosphoinositide-specific phospholipase C (PLC) superfamily of enzymes	N	N	N
70	PLEKHM1	Pleckstrin Homology And RUN Domain Containing M1	Essential for bone resorption, and may play a critical role in vesicular transport in the osteoclast	N	N	N
71	PLOD3	Procollagen-Lysine,2-Oxoglutarate 5-Dioxygenase 3	Membrane-bound homodimeric enzyme that catalyzes the hydroxylation of lysyl residues in collagen-like peptide	Y (Baek et al., 2018; Tsai et al., 2018; Baek et al., 2019; Xie et al., 2020)	N	Y (Shen et al., 2018)
72	PRSS3	Serine Protease 3	Member of the trypsin family of serine proteases.	Y (Hockla et al., 2012; Qian et al., 2017; Wang et al., 2019)	Putative (Marsit et al., 2005)	Y (Qian et al., 2017; Wang et al., 2019)
73	PSMA6	Proteasome 20S Subunit Alpha 6	Component of the 20S core proteasome complex involved in the proteolytic degradation of intracellular proteins	Y (Kakumu et al., 2017; Bakke et al., 2019)	N	N
74	PTPN23	Protein Tyrosine Phosphatase Non-Receptor Type 23	may be involved in the regulation of small nuclear ribonucleo protein assembly and pre-mRNA splicing	N	Y(Lin et al., 2011; Tanaka et al., 2013; Li et al., 2015; Li et al., 2019)	Y(Lin et al., 2011; Chen et al., 2020)
75	RAI1	Retinoic Acid Induced 1	Transcriptional regulator of the circadian clock components	N	N	N
76	RASA2	RAS P21 Protein Activator 2	Inhibitory regulator of the Ras-cyclic AMP pathway.	N	Y (Arafeh et al., 2015; Arafeh et al., 2019)	N
77	RASAL3	RAS Protein Activator Like 3	Functions as a Ras GTPase-activating protein	N	Putative (Mishra et al., 2018; Mishra et al., 2019)	N
78	REST	RE1 Silencing Transcription Factor	Transcriptional repressor which binds neuron-restrictive silencer element (NRSE) and represses neuronal gene transcription in non-neuronal cells	Y (Lawinger et al., 2000; Fuller et al., 2005; Huang et al., 2012; Negrini et al., 2013)	Y (Westbrook et al., 2005; Wagoner et al., 2010; Huang et al., 2012; Negrini et al., 2013)	N
79	RNF169	Ring Finger Protein 169	Probable E3 ubiquitin-protein ligase that acts as a negative regulator of double-strand breaks (DSBs) repair	N	N	Y (An et al., 2017; An et al., 2018)
80	RTDR1	Rhabdoid tumor deletion region 1	-	N	N	Y (Zhou et al., 2000)
81	SBNO1	Strawberry Notch Homolog 1	-	N	N	N
82	SERPINB13	Serpin Family B Member 13	A member of the serpin family of serine protease inhibitors, putative role in the proliferation/differentiation of keratinocytes	N	Y (Nakashima et al.., 2000; Martin et al., 2008)	Y (Kim et al., 2008; Sahu and Grandis, 2011)
83	SGMS2	Sphingomyelin Synthase 2	Synthesize the sphingolipid, sphingomyelin, through transfer of the phosphatidyl head group, phosphatidylcholine, on to the primary hydroxyl of ceramide	Y (Zheng et al., 2019)	N	N

84	SIDT1	SID1 Transmembrane Family Member 1	Role in RNA transport	N	Y (Wang et al., 2019)	Y (Wang et al., 2019)
85	SLC4A2	Solute Carrier Family 4 Member 2	Plasma membrane anion exchange protein	N	N	Y (Yang et al., 2008; Wang et al., 2013)
86	SPI1	Spi-1 Proto-Oncogene	Binds to PU-box, transcriptional activator specifically involved in the differentiation/activation of macrophages or B-cells.	Y (Moreau-Gachelin et al., 1988; Nguyen et al., 1990; Ray et al., 1990)	Y (Özdemir et al., 2018)	N
87	SRP9	Signal Recognition Particle 9	Crucial role in targeting secretory proteins to the rough endoplasmic reticulum membrane	N	N	Y (Rho et al., 2008)
88	STMN1	Stathmin 1	Microtubule destabilizer protein with an important role in cell cycle progression, cell proliferation, migration and survival.	Y (Chen et al., 2013; Zhang et al., 2020)	N	Y (Nemunaitis et al., 2012; Nie et al., 2015; Liu et al., 2017; Verdanet et al., 2017; Shimizu et al., 2019; Li et al., 2019)
89	STRN	Striatin	Calmodulin-binding protein which may function as scaffolding or signaling protein	N	N	Y (Kusano et al., 2016; Bastos et al., 2018Du et al., 2020)
90	TARP	TCR Gamma Alternate Reading Frame Protein	-	Putative (Wolfgang et al., 2000; Wolfgang et al., 2001)	N	Y (Fritzsche et al., 2010)
91	TBC1D16	TBC1 Domain Family Member 16	May act as a GTPase-activating protein for Rab family protein	Putative (Akavia et al., 2010; Vizoso et al., 2015; Rodger et al., 2019)	N	Y (Woouters et al., 2017; Yang et al., 2018)
92	TECTA	Tectorin Alpha	One of the major non-collagenous components of the tectorial membrane	N	N	Y (Hernández et al., 2007)
93	TGM1	Transglutaminase 1	Catalyzes the cross-linking of proteins and the conjugation of polyamines to proteins	N	N	Y (Martinet et al., 2003; Huang et al., 2017)
94	TIPRL	TOR Signaling Pathway Regulator	May be a allosteric regulator of serine/threonine-protein phosphatase 2A (PP2A)	N	Y (Luan et al., 2020)	Y (Song et al., 2012; Jeon et al., 2019; Jun et al., 2019; Xu t al., 2020)
95	TMEM54	Transmembrane Protein 54	-	N	N	N
96	TMPRSS11D	Transmembrane Serine Protease 11D	Trypsin-like serine protease released from the submucosal serous glands onto mucous membrane	Y (Cao et al., 2017; Yan et al., 2019)	N	Y (Cao et al., 2017; Zhao et al., 2019)
97	TRIM31	Tripartite Motif Containing 31	Functions as an E3 ubiquitin-protein ligase	Y (Sugiura and Miyamoto, 2008; Hatakeyama, 2011; Guo et al., 2018; Yu et al., 2018; Li et al., 2018; Zhou et al., 2019)	Y (Li et al., 2014)	Y (Yu et al., 2018)
98	USH1C	USH1 Protein Network Component Harmonin	Scaffold protein that functions in the assembly of Usher protein complexes	N	N	N
99	VIPR1	Vasoactive Intestinal Peptide Receptor 1	A receptor for vasoactive intestinal peptide, a small neuropeptide	N	Y(Mlakar et al., 2010; Zhao et al., 2019b)	Y (Yin et al., 2016; Sun et al., 2020)
100	WAS	WASP Actin Nucleation Promoting Factor	Effector protein for Rho-type GTPases that regulates actin filament reorganization	N	Y (Menotti et al., 2019)	Y (Ishihara et al., 2013)
101	ZDHHC12	Zinc Finger DHHC-Type Palmitoyltransferase 12	protein-cysteine S-palmitoyltransferase activity and palmitoyltransferase activity	N	N	Y (Chen et al., 2020; Meng et al., 2020)
102	ZDHHC20	Zinc Finger DHHC-Type Palmitoyltransferase 20	Catalyzes palmitoylation of Cys residues on target proteins	Y (Ducker et al., 2004; Draper and Smith, 2010; Jung et al., 2017)	N	N
103	ZNF814	zinc finger protein 814	Nucleic acid binding	N	N	Y (Karasaki et al., 2016; Ma et al., 2017)

Table S2 - Abbreviations used for the 31 different cancer types used in our analysis

Abbreviation	Cancer Type
LAML	acute myeloid leukaemia
ACC	adrenocortical carcinoma
BLCA	bladder urothelial carcinoma
LGG	brain lower grade glioma
BRCA	breast invasive carcinoma
CESC	cervical squamous cell carcinoma and endocervical adenocarcinoma
CHOL	cholangiocarcinoma
COAD	colon adenocarcinoma
UCEC	corpus endometrioid carcinoma
ESCA	oesophageal carcinoma
GBM	glioblastoma multiforme
HNSC	head & neck squamous cell carcinoma
KIRC	kidney renal clear cell carcinoma
KIRP	kidney renal papillary cell carcinoma
LIHC	liver hepatocellular carcinoma
LUAD	lung adenocarcinoma
LUSC	lung squamous cell carcinoma
DLBC	lymphoid neoplasm diffuse large B-cell lymphoma
MESO	Mesothelioma
OV	ovarian serous cystadenocarcinoma
PAAD	pancreatic adenocarcinoma
PCPG	pheochromocytoma and paraganglioma
PRAD	prostate adenocarcinoma
READ	rectum adenocarcinoma
SKCM	skin cutaneous melanoma
STAD	stomach adenocarcinoma
TGCT	testicular germ cell tumour
THYM	thymoma
THCA	thyroid carcinoma
UCS	uterine carcinosarcoma
UVM	uveal melanoma

Table S3 - Number of training and testing samples for each cancer type in our analysis

Cancer type	No. of Samples	
	Train	Test
LAML	138	35
ACC	63	16
BLCA	340	86
LGG	424	106
BRCA	974	244
CESC	246	62
CHOL	36	9
COAD	263	66
UCEC	160	41
ESCA	156	40
GBM	137	35
HNSC	452	114
KIRC	484	122
KIRP	258	65
LIHC	338	85
LUAD	460	116
LUSC	442	111
DLBC	38	10
MESO	69	18
OV	246	62
PAAD	146	37
PCPG	149	38
PRAD	440	110
READ	84	21
SKCM	379	95
STAD	360	90
TGCT	124	32
THYM	97	25
THCA	457	115
UCS	45	12
UVM	64	16

Table S4 - List of genes for each cancer type that showed combined precision and recall values greater than 0.75

Cancer Type	Genes
LAML	*TMEM54*
PRAD	*TARP*
LGG	*ATP1B2, ATPV1G2*
THCA	*FOXE1, PAX8*
KIRC	*PAX8, BHMT2, FASN*
LIHC	*MOSC2, HGD, DDR1*
BRCA	*DEFB132, CREB3L4, GDA, SIDT1, KLHL38*
KIRP	*KCP, RTDR1, GDA, C1orf21, CBFA2T3, ASNSD1*
STAD	*LCOR, EPS8L3, MUC13, TRIM31, ZDHHC20, PRSS3, EFNA2, GPN3, ASXL2, LATS1, PSMA6*
SKCM	*CABLES1, TBC1D16, PLOD3, VIPR1, SLC4A2, GOLGA2B, PTPN23, PLCH2, RAI1, HGD, SRP9, GJA1*

References

Aasen T, Leithe E, Graham SV, et al. Connexins in cancer: bridging the gap to the clinic. Oncogene. 2019;38(23):4429-4451. doi:10.1038/s41388-019-0741-6

Adam MA, Untch BR, Olson JA Jr. Parathyroid carcinoma: current understanding and new insights into gene expression and intraoperative parathyroid hormone kinetics. Oncologist. 2010;15(1):61-72. doi:10.1634/theoncologist.2009-0185

Akavia UD, Litvin O, Kim J, et al. An integrated approach to uncover drivers of cancer. Cell. 2010;143(6):1005-1017. doi:10.1016/j.cell.2010.11.013

Allison SJ. PAX8: a candidate oncogene in RCC. Nat Rev Nephrol. 2019;15(11):662. doi:10.1038/s41581-019-0205-9

An L, Dong C, Li J, et al. RNF169 limits 53BP1 deposition at DSBs to stimulate single-strand annealing repair. Proc Natl Acad Sci U S A. 2018;115(35):E8286-E8295. doi:10.1073/pnas.1804823115

An L, Jiang Y, Ng HH, et al. Dual-utility NLS drives RNF169-dependent DNA damage responses. Proc Natl Acad Sci U S A. 2017;114(14):E2872-E2881. doi:10.1073/pnas.1616602114

Arafeh R, Di Pizio A, Elkahloun AG, Dym O, Niv MY, Samuels Y. RASA2 and NF1; two-negative regulators of Ras with complementary functions in melanoma. Oncogene. 2019;38(13):2432-2434. doi:10.1038/s41388-018-0578-4

Arafeh R, Qutob N, Emmanuel R, et al. Recurrent inactivating RASA2 mutations in melanoma. Nat Genet. 2015;47(12):1408-1410. doi:10.1038/ng.3427

Arnason T, Pino MS, Yilmaz O, et al. Cables1 is a tumor suppressor gene that regulates intestinal tumor progression in Apc(Min) mice. Cancer Biol Ther. 2013;14(7):672-678. doi:10.4161/cbt.25089

Arora A, Abdel-Fatah TM, Agarwal D, et al. Transcriptomic and Protein Expression Analysis Reveals Clinicopathological Significance of Bloom Syndrome Helicase (BLM) in Breast Cancer. Mol Cancer Ther. 2015;14(4):1057-1065. doi:10.1158/1535-7163.MCT-14-0939

Baek JH, Yun HS, Kwon GT, et al. PLOD3 promotes lung metastasis via regulation of STAT3. Cell Death Dis. 2018;9(12):1138. Published 2018 Nov 15. doi:10.1038/s41419-018-1186-5

Baek JH, Yun HS, Kwon GT, et al. PLOD3 suppression exerts an anti-tumor effect on human lung cancer cells by modulating the PKC-delta signaling pathway. Cell Death Dis. 2019;10(3):156. Published 2019 Feb 15. doi:10.1038/s41419-019-1405-8

Bakke J, Wright WC, Zamora AE, et al. Genome-wide CRISPR screen reveals PSMA6 to be an essential gene in pancreatic cancer cells. BMC Cancer. 2019;19(1):253. Published 2019 Mar 21. doi:10.1186/s12885-019-5455-1

Bastos AU, de Jesus AC, Cerutti JM. ETV6-NTRK3 and STRN-ALK kinase fusions are recurrent events in papillary thyroid cancer of adult population. Eur J Endocrinol. 2018;178(1):83-91. doi:10.1530/EJE-17-0499

Bauer R, Ratzinger S, Wales L, et al. Inhibition of collagen XVI expression reduces glioma cell invasiveness. Cell Physiol Biochem. 2011;27(3-4):217-226. doi:10.1159/000327947

Berger MF, Lawrence MS, Demichelis F, et al. The genomic complexity of primary human prostate cancer. Nature. 2011;470(7333):214-220. doi:10.1038/nature09744

Brachtendorf S, El-Hindi K, Grösch S. Ceramide synthases in cancer therapy and chemoresistance. Prog Lipid Res. 2019;74:160-185. doi:10.1016/j.plipres.2019.04.002

Campregher PV, de Oliveira Pereira W, Lisboa B, et al. A novel mechanism of NPM1 cytoplasmic localization in acute myeloid leukemia: the recurrent gene fusion NPM1-HAUS1. Haematologica. 2016;101(7):e287-e290. doi:10.3324/haematol.2015.137364

Cao X, Tang Z, Huang F, Jin Q, Zhou X, Shi J. High TMPRSS11D protein expression predicts poor overall survival in non-small cell lung cancer. Oncotarget. 2017;8(8):12812-12819. doi:10.18632/oncotarget.14559

Castagnino A, Castro-Castro A, Irondelle M, et al. Coronin 1C promotes triple-negative breast cancer invasiveness through regulation of MT1-MMP traffic and invadopodia function. Oncogene. 2018;37(50):6425-6441. doi:10.1038/s41388-018-0422-x

Chen B, Pan Y, Xu X, et al. Inhibition of EPS8L3 suppresses liver cancer progression and enhances efficacy of sorafenib treatment. Biomed Pharmacother. 2020;128:110284. doi:10.1016/j.biopha.2020.110284

Chen D, Bhat-Nakshatri P, Goswami C, Badve S, Nakshatri H. ANTXR1, a stem cell-enriched functional biomarker, connects collagen signaling to cancer stem-like cells and metastasis in breast cancer. Cancer Res. 2013;73(18):5821-5833. doi:10.1158/0008-5472.CAN-13-1080

Chen J, Abi-Daoud M, Wang A, et al. Stathmin 1 is a potential novel oncogene in melanoma. Oncogene. 2013;32(10):1330-1337. doi:10.1038/onc.2012.141

Chen J, Zhao X, Yuan Y, Jing JJ. The expression patterns and the diagnostic/prognostic roles of PTPN family members in digestive tract cancers. Cancer Cell Int. 2020;20:238. Published 2020 Jun 12. doi:10.1186/s12935-020-01315-7

Chen L, Chan TH, Guan XY. Chromosome 1q21 amplification and oncogenes in hepatocellular carcinoma. Acta Pharmacol Sin. 2010;31(9):1165-1171. doi:10.1038/aps.2010.94

Chen WH, Cai MY, Zhang JX, et al. FMNL1 mediates nasopharyngeal carcinoma cell aggressiveness by epigenetically upregulating MTA1. Oncogene. 2018;37(48):6243-6258. doi:10.1038/s41388-018-0351-8

Chen X, Li H, Fan X, Zhao C, Ye K, Zhao Z, Hu L, Ma H, Wang H, Fang Z. Protein Palmitoylation Regulates Cell Survival by Modulating XBP1 Activity in Glioblastoma Multiforme. Molecular Therapy: Oncolytics 2020; 17:518-530.

Cheng X, Wang X, Wu Z, Tan S, Zhu T, Ding K. CORO1C expression is associated with poor survival rates in gastric cancer and promotes metastasis in vitro. FEBS Open Bio. 2019;9(6):1097-1108. doi:10.1002/2211-5463.12639

Cheng Y, Wang Y, Li J, Chang I, Wang CY. A novel read-through transcript JMJD7-PLA2G4B regulates head and neck squamous cell carcinoma cell proliferation and survival. Oncotarget. 2017;8(2):1972-1982. doi:10.18632/oncotarget.14081

Chiappetta G, Ammirante M, Basile A, et al. The antiapoptotic protein BAG3 is expressed in thyroid carcinomas and modulates apoptosis mediated by tumor necrosis factor-related apoptosis-inducing ligand. J Clin Endocrinol Metab. 2007;92(3):1159-1163. doi:10.1210/jc.2006-1712

Corominas-Faja B, Vellon L, Cuyàs E, et al. Clinical and therapeutic relevance of the metabolic oncogene fatty acid synthase in HER2+ breast cancer. Histol Histopathol. 2017;32(7):687-698. doi:10.14670/HH-11-830

Cowling VH, Turner SA, Cole MD. Burkitt's lymphoma-associated c-Myc mutations converge on a dramatically altered target gene response and implicate Nol5a/Nop56 in oncogenesis. Oncogene. 2014;33(27):3519-3527. doi:10.1038/onc.2013.338

Cruz MD, Wali RK, Bianchi LK, et al. Colonic mucosal fatty acid synthase as an early biomarker for colorectal neoplasia: modulation by obesity and gender. Cancer Epidemiol Biomarkers Prev. 2014;23(11):2413-2421. doi:10.1158/1055-9965.EPI-14-0026

Cui R, Yang L, Wang Y, et al. ASXL2 promotes colorectal cancer tumorigenesis by inducing cell proliferation. doi: 10.21203/rs.3.rs-25296/v1 (https://www.researchsquare.com/article/rs-25296/v1)

Dai W, Meng X, Mo S, et al. FOXE1 represses cell proliferation and Warburg effect by inhibiting HK2 in colorectal cancer. Cell Commun Signal. 2020;18(1):7. Published 2020 Jan 9. doi:10.1186/s12964-019-0502-8

Dai Z, Pendergast AM. Abi-2, a novel SH3-containing protein interacts with the c-Abl tyrosine kinase and modulates c-Abl transforming activity. Genes Dev. 1995;9(21):2569-2582. doi:10.1101/gad.9.21.2569

Dany M, Ogretmen B. Ceramide induced mitophagy and tumor suppression. Biochim Biophys Acta. 2015;1853(10 Pt B):2834-2845. doi:10.1016/j.bbamcr.2014.12.039

Daou S, Hammond-Martel I, Mashtalir N, et al. The BAP1/ASXL2 Histone H2A Deubiquitinase Complex Regulates Cell Proliferation and Is Disrupted in Cancer. J Biol Chem. 2015;290(48):28643-28663. doi:10.1074/jbc.M115.661553

de Voer RM, Hahn MM, Mensenkamp AR, et al. Deleterious Germline BLM Mutations and the Risk for Early-onset Colorectal Cancer. Sci Rep. 2015;5:14060. Published 2015 Sep 11. doi:10.1038/srep14060

de Winde CM, Veenbergen S, Young KH, et al. Tetraspanin CD37 protects against the development of B cell lymphoma. J Clin Invest. 2016;126(2):653-666. doi:10.1172/JCI81041

Demircan B, Dyer LM, Gerace M, Lobenhofer EK, Robertson KD, Brown KD. Comparative epigenomics of human and mouse mammary tumors. Genes Chromosomes Cancer. 2009;48(1):83-97. doi:10.1002/gcc.20620

Diquigiovanni C, Bergamini C, Evangelisti C, et al. Mutant MYO1F alters the mitochondrial network and induces tumor proliferation in thyroid cancer. Int J Cancer. 2018;143(7):1706-1719. doi:10.1002/ijc.31548

Draper JM, Smith CD. DHHC20: a human palmitoyl acyltransferase that causes cellular transformation. Mol Membr Biol. 2010;27(2-3):123-136. doi:10.3109/09687681003616854

Du QY, Yao JH, Zhou YC, Xu LJ, Zhao FY, Yang Y. High STRN Expression Promotes HCC Invasion and Migration but Not Cell Proliferation or Apoptosis through Facilitating Epithelial-Mesenchymal Transition. Biomed Res Int. 2020;2020:6152925. Published 2020 Mar 23. doi:10.1155/2020/6152925

Ducker CE, Stettler EM, French KJ, Upson JJ, Smith CD. Huntingtin interacting protein 14 is an oncogenic human protein: palmitoyl acyltransferase. Oncogene. 2004;23(57):9230-9237. doi:10.1038/sj.onc.1208171

Dufresne J, Bowden P, Thavarajah T, et al. The plasma peptides of breast versus ovarian cancer. Clin Proteomics. 2019;16:43. Published 2019 Dec 23. doi:10.1186/s12014-019-9262-0

Filippou PS, Ren AH, Korbakis D, et al. Exploring the potential of mucin 13 (MUC13) as a biomarker for carcinomas and other diseases. Clin Chem Lab Med. 2018;56(11):1945-1953. doi:10.1515/cclm-2018-0139

Fritzsche FR, Stephan C, Gerhardt J, et al. Diagnostic and prognostic value of T-cell receptor gamma alternative reading frame protein (TARP) expression in prostate cancer. Histol Histopathol. 2010;25(6):733-739. doi:10.14670/HH-25.733

Fukuda T, Guo L, Shi X, Wu C. CH-ILKBP regulates cell survival by facilitating the membrane translocation of protein kinase B/Akt. J Cell Biol. 2003;160(7):1001-1008. doi:10.1083/jcb.200212113

Fuller GN, Su X, Price RE, et al. Many human medulloblastoma tumors overexpress repressor element-1 silencing transcription (REST)/neuron-restrictive silencer factor, which can be functionally countered by REST-VP16. Mol Cancer Ther. 2005;4(3):343-349. doi:10.1158/1535-7163.MCT-04-0228

Furth N, Pateras IS, Rotkopf R, et al. LATS1 and LATS2 suppress breast cancer progression by maintaining cell identity and metabolic state. Life Sci Alliance. 2018;1(5):e201800171. Published 2018 Oct 30. doi:10.26508/lsa.201800171

Geng F, Lu GF, Ji MH, et al. MicroRNA-26b-3p/ANTXR1 signaling modulates proliferation, migration, and apoptosis of glioma. Am J Transl Res. 2019;11(12):7568-7578. Published 2019 Dec 15.

Gong J, Li Y, Liu CJ, et al. A Pan-cancer Analysis of the Expression and Clinical Relevance of Small Nucleolar RNAs in Human Cancer. Cell Rep. 2017;21(7):1968-1981. doi:10.1016/j.celrep.2017.10.070

Guey LT, García-Closas M, Murta-Nascimento C, et al. Genetic susceptibility to distinct bladder cancer subphenotypes. Eur Urol. 2010;57(2):283-292. doi:10.1016/j.eururo.2009.08.001

Guo P, Ma X, Zhao W, et al. TRIM31 is upregulated in hepatocellular carcinoma and promotes disease progression by inducing ubiquitination of TSC1-TSC2 complex. Oncogene. 2018;37(4):478-488. doi:10.1038/onc.2017.349

Guo WJ, Zeng MS, Yadav A, et al. Mel-18 acts as a tumor suppressor by repressing Bmi-1 expression and down-regulating Akt activity in breast cancer cells. Cancer Res. 2007;67(11):5083-5089. doi:10.1158/0008-5472.CAN-06-4368

Hannemann J, Velds A, Halfwerk JB, Kreike B, Peterse JL, van de Vijver MJ. Classification of ductal carcinoma in situ by gene expression profiling. Breast Cancer Res. 2006;8(5):R61. doi:10.1186/bcr1613

Hao Y, Chun A, Cheung K, Rashidi B, Yang X. Tumor suppressor LATS1 is a negative regulator of oncogene YAP. J Biol Chem. 2008;283(9):5496-5509. doi:10.1074/jbc.M709037200

Hatakeyama S. TRIM proteins and cancer. Nat Rev Cancer. 2011;11(11):792-804. Published 2011 Oct 7. doi:10.1038/nrc3139

Hernández P, Solé X, Valls J, et al. Integrative analysis of a cancer somatic mutome. Mol Cancer. 2007;6:13. Published 2007 Feb 5. doi:10.1186/1476-4598-6-13

Higa N, Shinsato Y, Kamil M, et al. Formin-like 1 (FMNL1) Is Associated with Glioblastoma Multiforme Mesenchymal Subtype and Independently Predicts Poor Prognosis. Int J Mol Sci. 2019;20(24):6355. Published 2019 Dec 17. doi:10.3390/ijms20246355

Hockla A, Miller E, Salameh MA, Copland JA, Radisky DC, Radisky ES. PRSS3/mesotrypsin is a therapeutic target for metastatic prostate cancer. Mol Cancer Res. 2012;10(12):1555-1566. doi:10.1158/1541-7786.MCR-12-0314

Hsu HM, Chu CM, Chang YJ, et al. Six novel immunoglobulin genes as biomarkers for better prognosis in triple-negative breast cancer by gene co-expression network analysis. Sci Rep. 2019;9(1):4484. Published 2019 Mar 14. doi:10.1038/s41598-019-40826-w

Hu Y, Li Z, Guo L, et al. MAGI-2 Inhibits cell migration and proliferation via PTEN in human hepatocarcinoma cells. Arch Biochem Biophys. 2007;467(1):1-9. doi:10.1016/j.abb.2007.07.027

Hu Y, Liu J, Jiang B, et al. MiR-199a-5p loss up-regulated DDR1 aggravated colorectal cancer by activating epithelial-to-mesenchymal transition related signaling. Dig Dis Sci. 2014;59(9):2163-2172. doi:10.1007/s10620-014-3136-0

Huang AH, Pan SH, Chang WH, Hong QS, Chen JJ, Yu SL. PARVA promotes metastasis by modulating ILK signalling pathway in lung adenocarcinoma. PLoS One. 2015;10(3):e0118530. Published 2015 Mar 4. doi:10.1371/journal.pone.0118530

Huang H, Chen Z, Ni X. Tissue transglutaminase-1 promotes stemness and chemoresistance in gastric cancer cells by regulating Wnt/β-catenin signaling. Exp Biol Med (Maywood). 2017;242(2):194-202. doi:10.1177/1535370216670541

Huang JR, Tan GM, Li Y, Shi Z. The Emerging Role of Cables1 in Cancer and Other Diseases. Mol Pharmacol. 2017;92(3):240-245. doi:10.1124/mol.116.107730

Huang T, She K, Peng G, et al. MicroRNA-186 suppresses cell proliferation and metastasis through targeting MAP3K2 in non-small cell lung cancer. Int J Oncol. 2016;49(4):1437-1444. doi:10.3892/ijo.2016.3637

Huang Z, Bao S. Ubiquitination and deubiquitination of REST and its roles in cancers. FEBS Lett. 2012;586(11):1602-1605. doi:10.1016/j.febslet.2012.04.052

Huo Y, Su T, Cai Q, Macara IG. An In Vivo Gain-of-Function Screen Identifies the Williams-Beuren Syndrome Gene GTF2IRD1 as a Mammary Tumor Promoter. Cell Rep. 2016;15(10):2089-2096. doi:10.1016/j.celrep.2016.05.011

Ishihara D, Dovas A, Hernandez L, et al. Wiskott-Aldrich syndrome protein regulates leukocyte-dependent breast cancer metastasis. Cell Rep. 2013;4(3):429-436. doi:10.1016/j.celrep.2013.07.007

Jalaguier S, Teyssier C, Nait Achour T, et al. Complex regulation of LCoR signaling in breast cancer cells. Oncogene. 2017;36(33):4790-4801. doi:10.1038/onc.2017.97

Jeon SJ, Ahn JH, Halder D, et al. TIPRL potentiates survival of lung cancer by inducing autophagy through the eIF2α-ATF4 pathway. Cell Death Dis. 2019;10(12):959. Published 2019 Dec 20. doi:10.1038/s41419-019-2190-0

Ji T, Liu D, Shao W, Yang W, Wu H, Bian X. Decreased expression of LATS1 is correlated with the progression and prognosis of glioma. J Exp Clin Cancer Res. 2012;31(1):67. Published 2012 Aug 21. doi:10.1186/1756-9966-31-67

Jiang S, Zhu L, Yang J, et al. Integrated expression profiling of potassium channels identifys KCNN4 as a prognostic biomarker of pancreatic cancer. Biochem Biophys Res Commun. 2017;494(1-2):113-119. doi:10.1016/j.bbrc.2017.10.072

Jun SY, Jeon SJ, Yoon JY, et al. The positive correlation of TIPRL with LC3 and CD133 contributes to cancer aggressiveness: potential biomarkers for early liver cancer. Sci Rep. 2019;9(1):16802. Published 2019 Nov 14. doi:10.1038/s41598-019-53191-5

Jung HC, Kim SH, Lee JH, Kim JH, Han SW. Gene Regulatory Network Analysis for Triple-Negative Breast Neoplasms by Using Gene Expression Data. J Breast Cancer. 2017;20(3):240-245. doi:10.4048/jbc.2017.20.3.240

Kakumu T, Sato M, Goto D, et al. Identification of proteasomal catalytic subunit PSMA6 as a therapeutic target for lung cancer. Cancer Sci. 2017;108(4):732-743. doi:10.1111/cas.13185

Kallel R, Belguith-Maalej S, Akdi A, et al. Genetic investigation of FOXE1 polyalanine tract in thyroid diseases: new insight on the role of FOXE1 in thyroid carcinoma. Cancer Biomark. 2010;8(1):43-51. doi:10.3233/DMA-2011-0824

Karasaki T, Nagayama K, Kawashima M, et al. Identification of Individual Cancer-Specific Somatic Mutations for Neoantigen-Based Immunotherapy of Lung Cancer. J Thorac Oncol. 2016;11(3):324-333. doi:10.1016/j.jtho.2015.11.006

Khan S, Sikander M, Ebeling MC, et al. MUC13 interaction with receptor tyrosine kinase HER2 drives pancreatic ductal adenocarcinoma progression. Oncogene. 2017;36(4):491-500. doi:10.1038/onc.2016.218

Khan S, Zafar N, Khan SS, et al. Clinical significance of MUC13 in pancreatic ductal adenocarcinoma. HPB (Oxford). 2018;20(6):563-572. doi:10.1016/j.hpb.2017.12.003

Kim TH, Park JM, Kim MY, Ahn YH. The role of CREB3L4 in the proliferation of prostate cancer cells. Sci Rep. 2017;7:45300. Published 2017 Mar 24. doi:10.1038/srep45300

Kim TJ, Choi JJ, Kim WY, et al. Gene expression profiling for the prediction of lymph node metastasis in patients with cervical cancer. Cancer Sci. 2008;99(1):31-38. doi:10.1111/j.1349-7006.2007.00652.x

Kochetkova M, McKenzie OL, Bais AJ, et al. CBFA2T3 (MTG16) is a putative breast tumor suppressor gene from the breast cancer loss of heterozygosity region at 16q24.3. Cancer Res. 2002;62(16):4599-4604.

Kong DH, Li S, Du ZX, et al. BAG3 elevation inhibits cell proliferation via direct interaction with G6PD in hepatocellular carcinomas. Oncotarget. 2016;7(1):700-711. doi:10.18632/oncotarget.6396

Kroll TG, Sarraf P, Pecciarini L, et al. PAX8-PPARgamma1 fusion oncogene in human thyroid carcinoma [corrected] [published correction appears in Science 2000 Sep 1;289(5484):1474]. Science. 2000;289(5483):1357-1360. doi:10.1126/science.289.5483.1357

Kuang Y, Wang Y, Zhai W, et al. Genome-Wide Analysis of Methylation-Driven Genes and Identification of an Eight-Gene Panel for Prognosis Prediction in Breast Cancer. Front Genet. 2020;11:301. Published 2020 Apr 21. doi:10.3389/fgene.2020.00301

Kumari S, Khan S, Gupta SC, et al. MUC13 contributes to rewiring of glucose metabolism in pancreatic cancer. Oncogenesis. 2018;7(2):19. Published 2018 Feb 22. doi:10.1038/s41389-018-0031-0

Kusano H, Togashi Y, Akiba J, et al. Two Cases of Renal Cell Carcinoma Harboring a Novel STRN-ALK Fusion Gene. Am J Surg Pathol. 2016;40(6):761-769. doi:10.1097/PAS.0000000000000610

Lara-Chacón B, Guerrero-Rodríguez SL, Ramírez-Hernández KJ, et al. Gpn3 Is Essential for Cell Proliferation of Breast Cancer Cells Independent of Their Malignancy Degree. Technol Cancer Res Treat. 2019;18:1533033819870823. doi:10.1177/1533033819870823

Laskowska J, Lewandowska-Bieniek J, Szczepanek J, Styczyński J, Tretyn A. Genomic and transcriptomic profiles and in vitro resistance to mitoxantrone and idarubicin in pediatric acute leukemias. J Gene Med. 2016;18(8):165-179. doi:10.1002/jgm.2889

Latini FR, Hemerly JP, Freitas BC, Oler G, Riggins GJ, Cerutti JM. ABI3 ectopic expression reduces in vitro and in vivo cell growth properties while inducing senescence. BMC Cancer. 2011;11:11. Published 2011 Jan 11. doi:10.1186/1471-2407-11-11

Lawinger P, Venugopal R, Guo ZS, et al. The neuronal repressor REST/NRSF is an essential regulator in medulloblastoma cells [published correction appears in Nat Med 2000 Sep;6(9):1062]. Nat Med. 2000;6(7):826-831. doi:10.1038/77565

Lee GY, Yang WI, Jeung HC, et al. Genome-wide genetic aberrations of thymoma using cDNA microarray based comparative genomic hybridization. BMC Genomics. 2007;8:305. Published 2007 Sep 3. doi:10.1186/1471-2164-8-305

Lee JY, Park MK, Park JH, et al. Loss of the polycomb protein Mel-18 enhances the epithelial-mesenchymal transition by ZEB1 and ZEB2 expression through the downregulation of miR-205 in breast cancer. Oncogene. 2014;33(10):1325-1335. doi:10.1038/onc.2013.53

Li D, Wang J, Zhang M, et al. LncRNA MAGI2-AS3 Is Regulated by BRD4 and Promotes Gastric Cancer Progression via Maintaining ZEB1 Overexpression by Sponging miR-141/200a. Mol Ther Nucleic Acids. 2020;19:109-123. doi:10.1016/j.omtn.2019.11.003

Li H, Zhang Y, Hai J, et al. Knockdown of TRIM31 suppresses proliferation and invasion of gallbladder cancer cells by down-regulating MMP2/9 through the PI3K/Akt signaling pathway. Biomed Pharmacother. 2018;103:1272-1278. doi:10.1016/j.biopha.2018.04.120

Li H, Zhang Y, Zhang Y, Bai X, Peng Y, He P. TRIM31 is downregulated in non-small cell lung cancer and serves as a potential tumor suppressor. Tumour Biol. 2014;35(6):5747-5752. doi:10.1007/s13277-014-1763-x

Li J, Qi Z, Hu YP, Wang YX. Possible biomarkers for predicting lymph node metastasis of esophageal squamous cell carcinoma: a review. J Int Med Res. 2019;47(2):544-556. doi:10.1177/0300060518819606

Li MY, Lai PL, Chou YT, et al. Protein tyrosine phosphatase PTPN3 inhibits lung cancer cell proliferation and migration by promoting EGFR endocytic degradation. Oncogene. 2015;34(29):3791-3803. doi:10.1038/onc.2014.312

Li MY, Peng WH, Wu CH, et al. PTPN3 suppresses lung cancer cell invasiveness by counteracting Src-mediated DAAM1 activation and actin polymerization. Oncogene. 2019;38(44):7002-7016. doi:10.1038/s41388-019-0948-6

Li P, Hu T, Wang H, et al. Upregulation of EPS8L3 is associated with tumorigenesis and poor prognosis in patients with liver cancer. Mol Med Rep. 2019;20(3):2493-2499. doi:10.3892/mmr.2019.10471

Li S, Dai Z, Yang D, et al. Targeting ß2 subunit of Na+/K+-ATPase induces glioblastoma cell apoptosis through elevation of intracellular Ca2. Am J Cancer Res. 2019;9(6):1293-1308. Published 2019 Jun 1.

Lin G, Aranda V, Muthuswamy SK, Tonks NK. Identification of PTPN23 as a novel regulator of cell invasion in mammary epithelial cells from a loss-of-function screen of the 'PTP-ome'. Genes Dev. 2011;25(13):1412-1425. doi:10.1101/gad.2018911

Link JM, Hurlin PJ. The activities of MYC, MNT and the MAX-interactome in lymphocyte proliferation and oncogenesis. Biochim Biophys Acta. 2015;1849(5):554-562. doi:10.1016/j.bbagrm.2014.04.004

Link JM, Ota S, Zhou ZQ, Daniel CJ, Sears RC, Hurlin PJ. A critical role for Mnt in Myc-driven T-cell proliferation and oncogenesis. Proc Natl Acad Sci U S A. 2012;109(48):19685-19690. doi:10.1073/pnas.1206406109

Liu H, Li Y, Li Y, Zhou L, Bie L. STMN1 as a candidate gene associated with atypical meningioma progression. Clin Neurol Neurosurg. 2017;159:107-110. doi:10.1016/j.clineuro.2017.06.003

Liu Z, Cao H, Shi Y, Yang R. KIAA1211 plays an oncogenic role in human non-small cell lung cancer. J Cancer. 2019;10(26):6747-6753. Published 2019 Oct 22. doi:10.7150/jca.35951

Luan M, Shi SS, Shi DB, et al. TIPRL, a Novel Tumor Suppressor, Suppresses Cell Migration, and Invasion Through Regulating AMPK/mTOR Signaling Pathway in Gastric Cancer. Front Oncol. 2020;10:1062. Published 2020 Jul 3. doi:10.3389/fonc.2020.01062

Luan XF, Wang L, Gai XF. The miR-28-5p-CAMTA2 axis regulates colon cancer progression via Wnt/ß-catenin signaling [published online ahead of print, 2019 Nov 10]. J Cell Biochem. 2019;10.1002/jcb.29536. doi:10.1002/jcb.29536

Ma D, Yang J, Wang Y, Huang X, Du G, Zhou L. Whole exome sequencing identified genetic variations in Chinese hemangioblastoma patients. Am J Med Genet A. 2017;173(10):2605-2613. doi:10.1002/ajmg.a.38350

Ma J, Huang X, Li Z, et al. FOXE1 supports the tumor promotion of Gli2 on papillary thyroid carcinoma by the Wnt/β-catenin pathway. J Cell Physiol. 2019;234(10):17739-17748. doi:10.1002/jcp.28399

Ma M, He M, Jiang Q, et al. MiR-487a Promotes TGF-β1-induced EMT, the Migration and Invasion of Breast Cancer Cells by Directly Targeting MAGI2. Int J Biol Sci. 2016;12(4):397-408. Published 2016 Feb 5. doi:10.7150/ijbs.13475

Mahdian R, Nodouzi V, Asgari M, et al. Expression profile of MAGI2 gene as a novel biomarker in combination with major deregulated genes in prostate cancer. Mol Biol Rep. 2014;41(9):6125-6131. doi:10.1007/s11033-014-3491-0

Marsit CJ, Okpukpara C, Danaee H, Kelsey KT. Epigenetic silencing of the PRSS3 putative tumor suppressor gene in non-small cell lung cancer. Mol Carcinog. 2005;44(2):146-150. doi:10.1002/mc.20125

Martin CL, Reshmi SC, Ried T, et al. Chromosomal imbalances in oral squamous cell carcinoma: examination of 31 cell lines and review of the literature. Oral Oncol. 2008;44(4):369-382. doi:10.1016/j.oraloncology.2007.05.003

Martinet N, Bonnard L, Regnault V, et al. In vivo transglutaminase type 1 expression in normal lung, preinvasive bronchial lesions, and lung cancer. Am J Respir Cell Mol Biol. 2003;28(4):428-435. doi:10.1165/rcmb.2002-0114OC

Masetti R, Bertuccio SN, Pession A, Locatelli F. CBFA2T3-GLIS2-positive acute myeloid leukaemia. A peculiar paediatric entity. *Br J Haematol* . 2019;184(3):337-347. doi:10.1111/bjh.15725

Mazur PK, Reynoird N, Khatri P, et al. SMYD3 links lysine methylation of MAP3K2 to Ras-driven cancer. Nature. 2014;510(7504):283-287. doi:10.1038/nature13320

Meng Y et al., ZDHHC12-mediated claudin-3S-palmitoylation determines ovarian cancer progression, Acta PharmaceuticaSinica B, 2020, https://doi.org/10.1016/j.apsb.2020.03.008

Menotti M, Ambrogio C, Cheong TC, et al. Wiskott-Aldrich syndrome protein (WASP) is a tumor suppressor in T cell lymphoma. Nat Med. 2019;25(1):130-140. doi:10.1038/s41591-018-0262-9

Micol JB, Pastore A, Inoue D, et al. ASXL2 is essential for haematopoiesis and acts as a haploinsufficient tumour suppressor in leukemia. Nat Commun. 2017;8:15429. Published 2017 May 18. doi:10.1038/ncomms15429

Milani L, Lundmark A, Kiialainen A, et al. DNA methylation for subtype classification and prediction of treatment outcome in patients with childhood acute lymphoblastic leukemia. Blood. 2010;115(6):1214-1225. doi:10.1182/blood-2009-04-214668

Mishra R, Haldar S, Placencio V, et al. Stromal epigenetic alterations drive metabolic and neuroendocrine prostate cancer reprogramming. J Clin Invest. 2018;128(10):4472-4484. doi:10.1172/JCI99397

Mishra R, Haldar S, Suchanti S, Bhowmick NA. Epigenetic changes in fibroblasts drive cancer metabolism and differentiation. Endocr Relat Cancer. 2019;26(12):R673-R688. doi:10.1530/ERC-19-0347

Mishra SK, Stephenson DJ, Chalfant CE, Brown RE. Upregulation of human glycolipid transfer protein (GLTP) induces necroptosis in colon carcinoma cells. Biochim Biophys Acta Mol Cell Biol Lipids. 2019;1864(2):158-167. doi:10.1016/j.bbalip.2018.11.002

Mlakar V, Strazisar M, Sok M, Glavac D. Oligonucleotide DNA microarray profiling of lung adenocarcinoma revealed significant downregulation and deletions of vasoactive intestinal peptide receptor 1. Cancer Invest. 2010;28(5):487-494. doi:10.3109/07357900903476752

Moiola C, De Luca P, Gardner K, Vazquez E, De Siervi A. Cyclin T1 overexpression induces malignant transformation and tumor growth. Cell Cycle. 2010;9(15):3119-3126. doi:10.4161/cc.9.15.12526

Moraes L, Galrão AL, Rubió I, Cerutti JM. Transcriptional regulation of the potential tumor suppressor ABI3 gene in thyroid carcinomas: interplay between methylation and NKX2-1 availability. Oncotarget. 2016;7(18):25960-25970. doi:10.18632/oncotarget.8416

Moreau-Gachelin F, Tavitian A, Tambourin P. Spi-1 is a putative oncogene in virally induced murine erythroleukaemias. Nature. 1988;331(6153):277-280. doi:10.1038/331277a0

Moro K, Kawaguchi T, Tsuchida J, et al. Ceramide species are elevated in human breast cancer and are associated with less aggressiveness. Oncotarget. 2018;9(28):19874-19890. Published 2018 Apr 13. doi:10.18632/oncotarget.24903

Nagpal JK, Dasgupta S, Jadallah S, et al. Profiling the expression pattern of GPI transamidase complex subunits in human cancer. Mod Pathol. 2008;21(8):979-991. doi:10.1038/modpathol.2008.76

Nakashima T, Pak SC, Silverman GA, Spring PM, Frederick MJ, Clayman GL. Genomic cloning, mapping, structure and promoter analysis of HEADPIN, a serpin which is down-regulated in head and neck cancer cells. Biochim Biophys Acta. 2000;1492(2-3):441-446. doi:10.1016/s0167-4781(00)00100-7

Nambara S, Masuda T, Kobayashi Y, et al. GTF2IRD1 on chromosome 7 is a novel oncogene regulating the tumor-suppressor gene TGFβR2 in colorectal cancer. Cancer Sci. 2020;111(2):343-355. doi:10.1111/cas.14248

Naser Al Deen N, AbouHaidar M, Talhouk R. Connexin43 as a Tumor Suppressor: Proposed Connexin43 mRNA-circularRNAs-microRNAs Axis Towards Prevention and Early Detection in Breast Cancer. Front Med (Lausanne). 2019;6:192. Published 2019 Aug 28. doi:10.3389/fmed.2019.00192

Negrini S, Prada I, D'Alessandro R, Meldolesi J. REST: an oncogene or a tumor suppressor?. Trends Cell Biol. 2013;23(6):289-295. doi:10.1016/j.tcb.2013.01.006

Nemunaitis J. Stathmin 1: a protein with many tasks. New biomarker and potential target in cancer. Expert Opin Ther Targets. 2012;16(7):631-634. doi:10.1517/14728222.2012.696101

Neumann LC, Weinhäusel A, Thomas S, Horsthemke B, Lohmann DR, Zeschnigk M. EFS shows biallelic methylation in uveal melanoma with poor prognosis as well as tissue-specific methylation. BMC Cancer. 2011;11:380. Published 2011 Aug 26. doi:10.1186/1471-2407-11-380

Nguyen VC, Ray D, Gross MS, de Tand MF, Frézal J, Moreau-Gachelin F. Localization of the human oncogene SPI1 on chromosome 11, region p11.22. Hum Genet. 1990;84(6):542-546. doi:10.1007/BF00210807

Nie W, Xu MD, Gan L, Huang H, Xiu Q, Li B. Overexpression of stathmin 1 is a poor prognostic biomarker in non-small cell lung cancer. Lab Invest. 2015;95(1):56-64. doi:10.1038/labinvest.2014.124

Nonaka D, Chiriboga L, Soslow RA. Expression of pax8 as a useful marker in distinguishing ovarian carcinomas from mammary carcinomas. Am J Surg Pathol. 2008;32(10):1566-1571. doi:10.1097/PAS.0b013e31816d71ad

Özdemir İ, Pınarlı FG, Pınarlı FA, et al. Epigenetic silencing of the tumor suppressor genes SPI1, PRDX2, KLF4, DLEC1, and DAPK1 in childhood and adolescent lymphomas. Pediatr Hematol Oncol. 2018;35(2):131-144. doi:10.1080/08880018.2018.1467986

Papadia C, Louwagie J, Del Rio P, et al. FOXE1 and SYNE1 genes hypermethylation panel as promising biomarker in colitis-associated colorectal neoplasia. Inflamm Bowel Dis. 2014;20(2):271-277. doi:10.1097/01.MIB.0000435443.07237.ed

Park UH, Kang MR, Kim EJ, et al. ASXL2 promotes proliferation of breast cancer cells by linking ERa to histone methylation. Oncogene. 2016;35(28):3742-3752. doi:10.1038/onc.2015.443

Payne M, Hickson ID. Genomic instability and cancer: lessons from analysis of Bloom's syndrome. Biochem Soc Trans. 2009;37(Pt 3):553-559. doi:10.1042/BST0370553

Peng Y, Wu D, Li F, Zhang P, Feng Y, He A. Identification of key biomarkers associated with cell adhesion in multiple myeloma by integrated bioinformatics analysis. Cancer Cell Int. 2020;20:262. Published 2020 Jun 22. doi:10.1186/s12935-020-01355-z

Piao XM, Jeong P, Yan C, et al. A novel tumor suppressing gene, ARHGAP9, is an independent prognostic biomarker for bladder cancer. Oncol Lett. 2020;19(1):476-486. doi:10.3892/ol.2019.11123

Pohl SÖ, Pervaiz S, Dharmarajan A, Agostino M. Gene expression analysis of heat-shock proteins and redox regulators reveals combinatorial prognostic markers in carcinomas of the gastrointestinal tract. Redox Biol. 2019;25:101060. doi:10.1016/j.redox.2018.11.018

Qian L, Gao X, Huang H, et al. PRSS3 is a prognostic marker in invasive ductal carcinoma of the breast. Oncotarget. 2017;8(13):21444-21453. doi:10.18632/oncotarget.15590

Ratzinger S, Grässel S, Dowejko A, Reichert TE, Bauer RJ. Induction of type XVI collagen expression facilitates proliferation of oral cancer cells. Matrix Biol. 2011;30(2):118-125. doi:10.1016/j.matbio.2011.01.001

Ray D, Culine S, Tavitain A, Moreau-Gachelin F. The human homologue of the putative proto-oncogene Spi-1: characterization and expression in tumors [published correction appears in Oncogene 1990 Oct;5(10):1611-2]. Oncogene. 1990;5(5):663-668.

Rho JH, Qin S, Wang JY, Roehrl MH. Proteomic expression analysis of surgical human colorectal cancer tissues: up-regulation of PSB7, PRDX1, and SRP9 and hypoxic adaptation in cancer. J Proteome Res. 2008;7(7):2959-2972. doi:10.1021/pr8000892

Ricklefs FL, Maire CL, Matschke J, et al. FASN Is a Biomarker Enriched in Malignant Glioma-Derived Extracellular Vesicles. Int J Mol Sci. 2020;21(6):1931. Published 2020 Mar 12. doi:10.3390/ijms21061931

Rodger EJ, Chatterjee A, Stockwell PA, Eccles MR. Characterisation of DNA methylation changes in EBF3 and TBC1D16 associated with tumour progression and metastasis in multiple cancer types. Clin Epigenetics. 2019;11(1):114. Published 2019 Aug 5. doi:10.1186/s13148-019-0710-5

Ross JB, Huh D, Noble LB, Tavazoie SF. Identification of molecular determinants of primary and metastatic tumour re-initiation in breast cancer. Nat Cell Biol. 2015;17(5):651-664. doi:10.1038/ncb3148

Sahu N, Grandis JR. New advances in molecular approaches to head and neck squamous cell carcinoma. Anticancer Drugs. 2011;22(7):656-664. doi:10.1097/CAD.0b013e32834249ba

Schaper F, van Spriel AB. Antitumor Immunity Is Controlled by Tetraspanin Proteins. Front Immunol. 2018;9:1185. Published 2018 May 29. doi:10.3389/fimmu.2018.01185

Schleifer RJ, Li S, Nechtman W, et al. KLHL5 knockdown increases cellular sensitivity to anticancer drugs. Oncotarget. 2018;9(100):37429-37438. Published 2018 Dec 21. doi:10.18632/oncotarget.26462

Senner V, Ratzinger S, Mertsch S, Grässel S, Paulus W. Collagen XVI expression is upregulated in glioblastomas and promotes tumor cell adhesion. FEBS Lett. 2008;582(23-24):3293-3300. doi:10.1016/j.febslet.2008.09.017

Sennoune SR, Bakunts K, Martínez GM, et al. Vacuolar H+-ATPase in human breast cancer cells with distinct metastatic potential: distribution and functional activity. Am J Physiol Cell Physiol. 2004;286(6):C1443-C1452. doi:10.1152/ajpcell.00407.2003

Sertkaya S, Hamid SM, Dilsiz N, Varisli L. Decreased expression of EFS is correlated with the advanced prostate cancer. Tumour Biol. 2015;36(2):799-805. doi:10.1007/s13277-014-2703-5

Shen Q, Eun JW, Lee K, et al. Barrier to autointegration factor 1, procollagen-lysine, 2-oxoglutarate 5-dioxygenase 3, and splicing factor 3b subunit 4 as early-stage cancer decision markers and drivers of hepatocellular carcinoma [published correction appears in Hepatology. 2018 Jul;68(1):391]. Hepatology. 2018;67(4):1360-1377. doi:10.1002/hep.29606

Sheng YH, He Y, Hasnain SZ, et al. MUC13 protects colorectal cancer cells from death by activating the NF-κB pathway and is a potential therapeutic target. Oncogene. 2017;36(5):700-713. doi:10.1038/onc.2016.241

Sheng YH, Wong KY, Seim I, et al. MUC13 promotes the development of colitis-associated colorectal tumors via β-catenin activity. Oncogene. 2019;38(48):7294-7310. doi:10.1038/s41388-019-0951-y

Shi H, Chen W, Dong Y, Lu X, Zhang W, Wang L. BAG3 promotes chondrosarcoma progression by upregulating the expression of β-catenin. Mol Med Rep. 2018;17(4):5754-5763. doi:10.3892/mmr.2018.8611

Shi X, Liu TT, Yu XN, et al. microRNA-93-5p promotes hepatocellular carcinoma progression via a microRNA-93-5p/MAP3K2/c-Jun positive feedback circuit [published online ahead of print, 2020 Jul 27]. Oncogene. 2020;10.1038/s41388-020-01401-0. doi:10.1038/s41388-020-01401-0

Shi Z, Li Z, Li ZJ, et al. Cables1 controls p21/Cip1 protein stability by antagonizing proteasome subunit alpha type 3. Oncogene. 2015;34(19):2538-2545. doi:10.1038/onc.2014.171

Shimizu K, Goto Y, Kawabata-Iwakawa R, et al. Stathmin-1 Is a Useful Diagnostic Marker for High-Grade Lung Neuroendocrine Tumors. Ann Thorac Surg. 2019;108(1):235-243. doi:10.1016/j.athoracsur.2019.02.040

Song IS, Jun SY, Na HJ, et al. Inhibition of MKK7-JNK by the TOR signaling pathway regulator-like protein contributes to resistance of HCC cells to TRAIL-induced apoptosis. Gastroenterology. 2012;143(5):1341-1351. doi:10.1053/j.gastro.2012.07.103

Song J, Chen X, Bai J, et al. Discoidin domain receptor 1 (DDR1), a promising biomarker, induces epithelial to mesenchymal transition in renal cancer cells. Tumour Biol. 2016;37(8):11509-11521. doi:10.1007/s13277-016-5021-2

Sugimachi K, Matsumura T, Shimamura T, et al. Aberrant Methylation of FOXE1 Contributes to a Poor Prognosis for Patients with Colorectal Cancer. Ann Surg Oncol. 2016;23(12):3948-3955. doi:10.1245/s10434-016-5289-x

Sugiura T, Miyamoto K. Characterization of TRIM31, upregulated in gastric adenocarcinoma, as a novel RBCC protein. J Cell Biochem. 2008;105(4):1081-1091. doi:10.1002/jcb.21908

Sun L, Zhang Z, Yao Y, Li WY, Gu J. Analysis of expression differences of immune genes in non-small cell lung cancer based on TCGA and ImmPort data sets and the application of a prognostic model. Ann Transl Med. 2020;8(8):550. doi:10.21037/atm.2020.04.38

Takai K, Drain AP, Lawson DA, et al. Discoidin domain receptor 1 (DDR1) ablation promotes tissue fibrosis and hypoxia to induce aggressive basal-like breast cancers. Genes Dev. 2018;32(3-4):244-257. doi:10.1101/gad.301366.117

Taki T, Akiyama M, Saito S, et al. The MYO1F, unconventional myosin type 1F, gene is fused to MLL in infant acute monocytic leukemia with a complex translocation involving chromosomes 7, 11, 19 and 22. Oncogene. 2005;24(33):5191-5197. doi:10.1038/sj.onc.1208711

Talhouk RS, Fares MB, Rahme GJ, et al. Context dependent reversion of tumor phenotype by connexin-43 expression in MDA-MB231 cells and MCF-7 cells: role of β-catenin/connexin43 association. Exp Cell Res. 2013;319(20):3065-3080. doi:10.1016/j.yexcr.2013.10.002

Tanaka K, Kondo K, Kitajima K, Muraoka M, Nozawa A, Hara T. Tumor-suppressive function of protein-tyrosine phosphatase non-receptor type 23 in testicular germ cell tumors is lost upon overexpression of miR142-3p microRNA. J Biol Chem. 2013;288(33):23990-23999. doi:10.1074/jbc.M113.478891

Teleki I, Szasz AM, Maros ME, et al. Correlations of differentially expressed gap junction connexins Cx26, Cx30, Cx32, Cx43 and Cx46 with breast cancer progression and prognosis. PLoS One. 2014;9(11):e112541. Published 2014 Nov 10. doi:10.1371/journal.pone.0112541

Terrasi A, Bertolini I, Martelli C, et al. Specific V-ATPase expression sub-classifies IDHwt lower-grade gliomas and impacts glioma growth in vivo. EBioMedicine. 2019;41:214-224. doi:10.1016/j.ebiom.2019.01.052

Thomas M, Laura R, Hepner K, et al. Oncogenic human papillomavirus E6 proteins target the MAGI-2 and MAGI-3 proteins for degradation. Oncogene. 2002;21(33):5088-5096. doi:10.1038/sj.onc.1205668

Tiemin P, Fanzheng M, Peng X, et al. MUC13 promotes intrahepatic cholangiocarcinoma progression via EGFR/PI3K/AKT pathways. J Hepatol. 2020;72(4):761-773. doi:10.1016/j.jhep.2019.11.021

Toth R, Schiffmann H, Hube-Magg C, et al. Random forest-based modelling to detect biomarkers for prostate cancer progression. Clin Epigenetics. 2019;11(1):148. Published 2019 Oct 22. doi:10.1186/s13148-019-0736-8

Triki M, Ben Ayed-Guerfali D, Saguem I, et al. RIP140 and LCoR expression in gastrointestinal cancers. Oncotarget. 2017;8(67):111161-111175. Published 2017 Nov 25. doi:10.18632/oncotarget.22686

Tsai CK, Huang LC, Tsai WC, Huang SM, Lee JT, Hueng DY. Overexpression of PLOD3 promotes tumor progression and poor prognosis in gliomas. Oncotarget. 2018;9(21):15705-15720. Published 2018 Feb 28. doi:10.18632/oncotarget.24594

Valiathan RR, Marco M, Leitinger B, Kleer CG, Fridman R. Discoidin domain receptor tyrosine kinases: new players in cancer progression. Cancer Metastasis Rev. 2012;31(1-2):295-321. doi:10.1007/s10555-012-9346-z

Valle BL, Rodriguez-Torres S, Kuhn E, et al. HIST1H2BB and MAGI2 Methylation and Somatic Mutations as Precision Medicine Biomarkers for Diagnosis and Prognosis of High-grade Serous Ovarian Cancer [published online ahead of print, 2020 Jun 24]. Cancer Prev Res (Phila). 2020;10.1158/1940-6207.CAPR-19-0412. doi:10.1158/1940-6207.CAPR-19-0412

van den Boom J, Wolter M, Blaschke B, Knobbe CB, Reifenberger G. Identification of novel genes associated with astrocytoma progression using suppression subtractive hybridization and real-time reverse transcription-polymerase chain reaction. Int J Cancer. 2006;119(10):2330-2338. doi:10.1002/ijc.22108

Velazquez-Torres G, Shoshan E, Ivan C, et al. A-to-I miR-378a-3p editing can prevent melanoma progression via regulation of PARVA expression. Nat Commun. 2018;9(1):461. Published 2018 Jan 31. doi:10.1038/s41467-018-02851-7

Verdanet E, Dereure O, René C, et al. Diagnostic value of STMN1, LMO2, HGAL, AID expression and 1p36 chromosomal abnormalities in primary cutaneous B cell lymphomas. Histopathology. 2017;71(4):648-660. doi:10.1111/his.13279

Visca P, Sebastiani V, Botti C, et al. Fatty acid synthase (FAS) is a marker of increased risk of recurrence in lung carcinoma. *Anticancer Res* . 2004;24(6):4169-4173.

Vizoso M, Ferreira HJ, Lopez-Serra P, et al. Epigenetic activation of a cryptic TBC1D16 transcript enhances melanoma progression by targeting EGFR. Nat Med. 2015;21(7):741-750. doi:10.1038/nm.3863

Vogelsang TLR, Schmoeckel E, Kuhn C, et al. Regulation of LCoR and RIP140 expression in cervical intraepithelial neoplasia and correlation with CIN progression and dedifferentiation. J Cancer Res Clin Oncol. 2020;146(7):1847-1855. doi:10.1007/s00432-020-03178-x

Wagoner MP, Gunsalus KT, Schoenike B, Richardson AL, Friedl A, Roopra A. The transcription factor REST is lost in aggressive breast cancer. PLoS Genet. 2010;6(6):e1000979. Published 2010 Jun 10. doi:10.1371/journal.pgen.1000979

Walts AE, Said JW, Siegel MB, Banks-Schlegel S. Involucrin, a marker of squamous and urothelial differentiation. An immunohistochemical study on its distribution in normal and neoplastic tissues. J Pathol. 1985;145(4):329-340. doi:10.1002/path.1711450406

Wang C, Yang Y, Yin L, et al. Novel Potential Biomarkers Associated With Epithelial to Mesenchymal Transition and Bladder Cancer Prognosis Identified by Integrated Bioinformatic Analysis. Front Oncol. 2020;10:931. Published 2020 Jun 30. doi:10.3389/fonc.2020.00931

Wang F, Hu YL, Feng Y, et al. High-level expression of PRSS3 correlates with metastasis and poor prognosis in patients with gastric cancer. J Surg Oncol. 2019;119(8):1108-1121. doi:10.1002/jso.25448

Wang LL, Zhang M. miR-582-5p is a potential prognostic marker in human non-small cell lung cancer and functions as a tumor suppressor by targeting MAP3K2. Eur Rev Med Pharmacol Sci. 2018;22(22):7760-7767. doi:10.26355/eurrev_201811_16397

Wang M, Ma H, Pan Y, et al. PAX2 and PAX8 reliably distinguishes ovarian serous tumors from mucinous tumors. Appl Immunohistochem Mol Morphol. 2015;23(4):280-287. doi:10.1097/PAI.0000000000000065

Wang T, Zhao L, Yang Y, et al. EGR1 is critical for gastrin-dependent upregulation of anion exchanger 2 in gastric cancer cells. FEBS J. 2013;280(1):174-183. doi:10.1111/febs.12058

Wang Y, Li H, Ma J, et al. Integrated Bioinformatics Data Analysis Reveals Prognostic Significance Of SIDT1 In Triple-Negative Breast Cancer. Onco Targets Ther. 2019;12:8401-8410. Published 2019 Oct 11. doi:10.2147/OTT.S215898

Wang Y, Wang Y, Li J, et al. PAX8: a sensitive and specific marker to identify cancer cells of ovarian origin for patients prior to neoadjuvant chemotherapy. J Hematol Oncol. 2013;6:60. Published 2013 Aug 19. doi:10.1186/1756-8722-6-60

Westbrook TF, Martin ES, Schlabach MR, et al. A genetic screen for candidate tumor suppressors identifies REST. Cell. 2005;121(6):837-848. doi:10.1016/j.cell.2005.03.033

Wolfgang CD, Essand M, Lee B, Pastan I. T-cell receptor gamma chain alternate reading frame protein (TARP) expression in prostate cancer cells leads to an increased growth rate and induction of caveolins and amphiregulin. Cancer Res. 2001;61(22):8122-8126.

Wolfgang CD, Essand M, Vincent JJ, Lee B, Pastan I. TARP: a nuclear protein expressed in prostate and breast cancer cells derived from an alternate reading frame of the T cell receptor gamma chain locus. Proc Natl Acad Sci U S A. 2000;97(17):9437-9442. doi:10.1073/pnas.160270597

Wouters J, Vizoso M, Martinez-Cardus A, et al. Comprehensive DNA methylation study identifies novel progression-related and prognostic markers for cutaneous melanoma. BMC Med. 2017;15(1):101. Published 2017 Jun 5. doi:10.1186/s12916-017-0851-3

Wu D, Wang Y, Yang G, et al. A novel mitochondrial amidoxime reducing component 2 is a favorable indicator of cancer and suppresses the progression of hepatocellular carcinoma by regulating the expression of p27 [published online ahead of print, 2020 Aug 18]. Oncogene. 2020;10.1038/s41388-020-01417-6. doi:10.1038/s41388-020-01417-6

Wu G, Guo Z, Chatterjee A, et al. Overexpression of glycosylphosphatidylinositol (GPI) transamidase subunits phosphatidylinositol glycan class T and/or GPI anchor attachment 1 induces tumorigenesis and contributes to invasion in human breast cancer. Cancer Res. 2006;66(20):9829-9836. doi:10.1158/0008-5472.CAN-06-0506

Wu L, Peng CW, Hou JX, et al. Coronin-1C is a novel biomarker for hepatocellular carcinoma invasive progression identified by proteomics analysis and clinical validation. J Exp Clin Cancer Res. 2010;29(1):17. Published 2010 Feb 24. doi:10.1186/1756-9966-29-17

Xia H, Qi H, Li Y, et al. LATS1 tumor suppressor regulates G2/M transition and apoptosis. Oncogene. 2002;21(8):1233-1241. doi:10.1038/sj.onc.1205174

Xiang L, Kong B. PAX8 is a novel marker for differentiating between various types of tumor, particularly ovarian epithelial carcinomas. Oncol Lett. 2013;5(3):735-738. doi:10.3892/ol.2013.1121

Xie D, Li J, Wei S, et al. Knockdown of PLOD3 suppresses the malignant progression of renal cell carcinoma via reducing TWIST1 expression [published online ahead of print, 2020 Jun 22]. Mol Cell Probes. 2020;101608. doi:10.1016/j.mcp.2020.101608

Xu K, Mao X, Mehta M, et al. Elucidation of how cancer cells avoid acidosis through comparative transcriptomic data analysis. PLoS One. 2013;8(8):e71177. Published 2013 Aug 14. doi:10.1371/journal.pone.0071177

Xu X, Zhu H, Yang M, et al. Knockdown of TOR signaling pathway regulator suppresses cell migration and invasion in non-small cell lung cancer via the regulation of epithelial-to-mesenchymal transition. Exp Ther Med. 2020;19(3):1925-1932. doi:10.3892/etm.2019.8358

Xu Y, Wu G, Li J, et al. Screening and Identification of Key Biomarkers for Bladder Cancer: A Study Based on TCGA and GEO Data. Biomed Res Int. 2020;2020:8283401. Published 2020 Jan 23. doi:10.1155/2020/8283401

Xuan Z, Zhao L, Li Z, et al. EPS8L3 promotes hepatocellular carcinoma proliferation and metastasis by modulating EGFR dimerization and internalization. Am J Cancer Res. 2020;10(1):60-77. Published 2020 Jan 1.

Yan HX, Yang W, Zhang R, et al. Protein-tyrosine phosphatase PCP-2 inhibits beta-catenin signaling and increases E-cadherin-dependent cell adhesion. J Biol Chem. 2006;281(22):15423-15433. doi:10.1074/jbc.M602607200

Yan K, Hu C, Liu C, Chu G, Wang X, Mac S, Li L. Knockdown of TMPRSS11D inhibits the proliferation, migration and invasion of cervical cancer cells. RSC Adv., 2019; 9:21591

Yang X, Yu K, Hao Y, et al. LATS1 tumour suppressor affects cytokinesis by inhibiting LIMK1. Nat Cell Biol. 2004;6(7):609-617. doi:10.1038/ncb1140

Yang Y, Wu PP, Wu J, et al. Expression of anion exchanger 2 in human gastric cancer. Exp Oncol. 2008;30(1):81-87.

Yang Z, Shen H, He W, et al. Expression of TBC1D16 Is Associated with Favorable Prognosis of Epithelial Ovarian Cancer. Tohoku J Exp Med. 2018;245(3):141-148. doi:10.1620/tjem.245.141

Yin F, Shu L, Liu X, et al. Microarray-based identification of genes associated with cancer progression and prognosis in hepatocellular carcinoma. J Exp Clin Cancer Res. 2016;35(1):127. Published 2016 Aug 27. doi:10.1186/s13046-016-0403-2

Yoshihara K, Wang Q, Torres-Garcia W, et al. The landscape and therapeutic relevance of cancer-associated transcript fusions. Oncogene. 2015;34(37):4845-4854. doi:10.1038/onc.2014.406

Yu C, Chen S, Guo Y, Sun C. Oncogenic TRIM31 confers gemcitabine resistance in pancreatic cancer via activating the NF-κB signaling pathway. Theranostics. 2018;8(12):3224-3236. Published 2018 May 11. doi:10.7150/thno.23259

Zakrzewska M, Zakrzewski K, Grešner SM, Piaskowski S, Zalewska-Szewczyk B, Liberski PP. Polycomb genes expression as a predictor of poor clinical outcome in children with medulloblastoma. Childs Nerv Syst. 2011;27(1):79-86. doi:10.1007/s00381-010-1260-5

Zeng CX, Tang LY, Xie CY, et al. Overexpression of EPS8L3 promotes cell proliferation by inhibiting the transactivity of FOXO1 in HCC. Neoplasma. 2018;65(5):701-707. doi:10.4149/neo_2018_170725N503

Zhang Q, Han Q, Zi J, Song C, Ge Z. CD37 high expression as a potential biomarker and association with poor outcome in acute myeloid leukemia. Biosci Rep. 2020;40(5):BSR20200008. doi:10.1042/BSR20200008

Zhang R, Gao X, Zuo J, et al. STMN1 upregulation mediates hepatocellular carcinoma and hepatic stellate cell crosstalk to aggravate cancer by triggering the MET pathway. Cancer Sci. 2020;111(2):406-417. doi:10.1111/cas.14262

Zhao L, Chi W, Cao H, et al. Screening and clinical significance of tumor markers in head and neck squamous cell carcinoma through bioinformatics analysis. Mol Med Rep. 2019;19(1):143-154. doi:10.3892/mmr.2018.9639

Zhao L, Yu Z, Zhao B. Mechanism of VIPR1 gene regulating human lung adenocarcinoma H1299 cells. Med Oncol. 2019b;36(11):91. Published 2019 Sep 27. doi:10.1007/s12032-019-1312-y

Zheng K, Chen Z, Feng H, et al. Sphingomyelin synthase 2 promotes an aggressive breast cancer phenotype by disrupting the homoeostasis of ceramide and sphingomyelin. Cell Death Dis. 2019;10(3):157. Published 2019 Feb 15. doi:10.1038/s41419-019-1303-0

Zhou J, Fogelgren B, Wang Z, Roe BA, Biegel JA. Isolation of genes from the rhabdoid tumor deletion region in chromosome band 22q11.2. Gene. 2000;241(1):133-141. doi:10.1016/s0378-1119(99)00456-4

Zhou L, Deng ZZ, Li HY, et al. TRIM31 promotes glioma proliferation and invasion through activating NF-κB pathway. Onco Targets Ther. 2019;12:2289-2297. Published 2019 Mar 27. doi:10.2147/OTT.S183625

Zhou M, Fan J, Li Z, et al. Prognostic impact of tumor mutation burden and the mutation in KIAA1211 in small cell lung cancer. Respir Res. 2019;20(1):248. Published 2019 Nov 7. doi:10.1186/s12931-019-1205-9
